# Species-specific PHYTOCHROME-INTERACTING FACTOR utilization in the plant morphogenetic response to environmental stimuli

**DOI:** 10.1093/plcell/koaf048

**Published:** 2025-03-14

**Authors:** Srinivas Kunta, Yardena Dahan, Shai Torgeman, Joanne Chory, Yogev Burko

**Affiliations:** The Institute of Plant Sciences, Agricultural Research Organization, Volcani Center, Rishon LeZion 7505101, Israel; The Institute of Plant Sciences, Agricultural Research Organization, Volcani Center, Rishon LeZion 7505101, Israel; Institute of Plant Science and Genetics in Agriculture, The Robert H. Smith Faculty of Agriculture, Food and Environment, The Hebrew University of Jerusalem, Rehovot 76100, Israel; Howard Hughes Medical Institute, Salk Institute for Biological Studies, La Jolla, CA 92037, USA; Plant Biology Laboratory, Salk Institute for Biological Studies, 10010 North Torrey Pines Road, La Jolla, CA 92037, USA; The Institute of Plant Sciences, Agricultural Research Organization, Volcani Center, Rishon LeZion 7505101, Israel

## Abstract

PHYTOCHROME-INTERACTING FACTORs (PIFs) regulate growth-related gene expression in response to environmental conditions. Among their diverse functions in regulating signal responses, PIFs play an important role in thermomorphogenesis (the response to increased ambient temperature) and in the shade avoidance response. While numerous studies have examined the varied roles of PIFs in Arabidopsis (*Arabidopsis thaliana*), their roles in crop plants remain poorly investigated. This study delves into the conservation of PIFs activity among species by examining their functions in tomato (*Solanum lycopersicum*) and comparing them to known PIF functions in Arabidopsis using single and higher-order mutants of tomato *PIF* genes (*SlPIF*s). We demonstrate that, in contrast to Arabidopsis, PIFs are not required for thermomorphogenesis-induced stem elongation in tomato. In addition, whereas Arabidopsis PIF8 has a minor effect on plant growth, tomato SlPIF8a plays a key role in the low red/far-red (R/FR) response. In contrast, SlPIF4 and SlPIF7*s* play minor roles in this process. We also investigated the tissue-specific low R/FR response in tomato seedlings and demonstrate that the aboveground organs exhibit a conserved response to low R/FR, which is regulated by SlPIFs. Our findings provide insights into PIF-mediated responses in crop plants, which may guide future breeding strategies to enhance yield under high planting densities.

## Teaser

Specific PHYTOCHROME-INTERACTING FACTOR members regulate shade avoidance in Arabidopsis and tomato but are not required for thermomorphogenesis-induced stem elongation in tomato.

## Introduction

In agricultural settings, plants of the same species (monocultures) are usually grown at relatively high densities. This practice promotes competition for resources among neighboring plants, both above and below ground. The close proximity of neighboring plants associated with high plant densities limits the availability of light to drive photosynthesis, intensifying the importance of the competition for light. Most sun-loving plants have evolved adaptive strategies to escape from the shade cast by their neighbors through profound changes in their morphology and physiology (Morgan and Smith 1976; Morgan and Smith 1979; Villalobos et al. 1994; Libenson et al. 2002; Ballaré et al. 1997). This phenomenon, known as the shade avoidance syndrome (SAS), includes stem elongation, leaf hyponasty, inhibition of leaf expansion, adjustments in photosynthetic metabolism, and earlier initiation of the reproductive stage of development (Smith 1982; Neff et al. 2000; Libenson et al. 2002; Smith and Whitelam 1997; Casal 2012; Casal and Fankhauser 2023). SAS is initiated by photoreceptors, primarily phytochrome B (phyB), which senses changes in the ratio of red to far-red (R/FR) light. FR light reflected from neighboring leaves causes a reduction in the R/FR ratio, enabling the phyB in a particular plant to detect other nearby plants before that plant becomes fully shaded by its neighbors (i.e. neighbor detection; Ballaré et al. 1990, 2006). Mechanical signals derived from physical contact with the leaves of neighboring plants also contribute to the full SAS (de Wit et al. 2012).

Under high R/FR conditions, such as in direct sunlight, phyB is active (Pfr) and translocates to the nucleus, where it physically interacts with the bHLH transcription factors known as PHYTOCHROME-INTERACTING FACTORS (PIFs), causing their inactivation and degradation (Park et al. 2004; Al-Sady et al. 2006; Lorrain et al. 2008; Shen et al. 2008; Leivar and Quail 2011; Li et al. 2012). When the R/FR drops below1, as planting density increases, phyB is photoconverted into its inactive form (Pr). This inactivation releases PIFs from the phyB repression, enabling them to bind to their target gene promoters and regulate their expression (Franklin 2008; Lorrain et al. 2008; Franklin and Quail 2010; Casal 2013; Legris et al. 2019). phyB was also found to be able to sense temperature. In this scenario, warm temperatures promote the thermal reversion of phyB from its active form to its inactive form (Jung et al. 2016; Legris et al. 2016; Sellaro et al. 2019). This inactivation also releases PIFs from phyB repression, allowing them to regulate the transcription of growth-related genes, which promotes the thermomorphogenesis response.

The PIFs’ downstream target genes are associated with auxin, gibberellin (GA), and cell wall remodeling, which together promote growth (Oh et al. 2007; Hornitschek et al. 2012; Li et al. 2012; Leivar and Monte 2014; Filo et al. 2015; Mizuno et al. 2015; Pedmale et al. 2016; Krahmer and Fankhauser 2023). Arabidopsis (*Arabidopsis thaliana*) possesses at least 8 PIFs (PIF1 to PIF8), which regulate different aspects of the plant's responses to light and temperature (Leivar and Quail 2011; Jeong and Choi 2013; Pham et al. 2018). The dominant PIF controlling the low R/FR response is PIF7, with minor contributions from PIF1, PIF3, PIF4, and PIF5, whereas no detectable role has been observed for PIF8 (Lorrain et al. 2008; Leivar et al. 2012; Li et al. 2012; Oh et al. 2020). During the thermomorphogenesis response, PIF4 and PIF7 predominantly regulate the warm temperature-induced hypocotyl elongation (Koini et al. 2009; Raschke et al. 2015; Chung et al. 2020; Fiorucci et al. 2020; Kim et al. 2020; Bianchimano et al. 2023; Quint et al. 2023). Low R/FR and thermomorphogenesis are initiated by the binding of PIF7 and PIF4 to the promoters of auxin biosynthesis genes in the cotyledons (Franklin et al. 2011; Hornitschek et al. 2012). Subsequently, auxin travels to the hypocotyl and promotes hypocotyl elongation (Estelle 1998; Pierik et al. 2009; Keuskamp et al. 2010; Kozuka et al. 2010; Procko et al. 2014; Kohnen et al. 2016; Bellstaedt et al. 2019; Bianchimano et al. 2023). Recent studies that combined shade (low R/FR) with warm temperatures to mimic natural environments revealed that warm temperatures further enhance the elongation induced by shade (Weinig 2000; Romero-Montepaone et al. 2020; Romero-Montepaone et al. 2021; Burko et al. 2022; Casal and Fankhauser 2023). This interaction between shade and temperature is primarily regulated by PIF7 (Burko et al. 2022).

Tomato (*Solanum lycopersicum*) also possesses 8 PIFs: *SlPIF1a*, *SlPIF1b*, *SlPIF3*, *SlPIF4*, *SlPIF7a*, *SlPIF7b*, *SlPIF8a*, and *SlPIF8b*, which are homologous to 5 of the 8 Arabidopsis PIFs, with gene duplications occurring in *SlPIF1*, *SlPIF7*, and *SlPIF8* (Rosado et al. 2016). *SlPIF1a* and *SlPIF1b* have been shown to regulate seed germination, pigment biosynthesis, root hair elongation, flowering time, fruit growth, and fruit metabolism (Simon-Moya et al. 2021). *SlPIF1a* and *SlPIF3* regulate fruit metabolism in a light-dependent manner (Llorente et al. 2016; Gramegna et al. 2019), and *SlPIF4* regulates cold tolerance, fruit ripening, carotenoid levels, flowering time, fruit yield, and fruit size (Rosado et al. 2019; Wang et al. 2020; Pan et al. 2021). While tomato responses to low R/FR have been observed in the shoot and in the roots, the role of SlPIFs in these responses is poorly understood (Cagnola et al. 2012; Chitwood et al. 2015; Schrager-Lavelle et al. 2016; Rosado et al. 2022). SlPIF4 has been shown to play a role in regulating tomato hypocotyl elongation in young seedlings grown on media plates (Rosado et al. 2019; Sun et al. 2020; Zhu et al. 2024). However, evidence is mixed regarding the growth conditions under which SlPIF4 plays a role in regulating hypocotyl elongation. Sun et al. (2020) found that both *SlPIF4* mutants and overexpression lines displayed impaired hypocotyl elongation when grown under either continuous white light or continuous simulated shade (composed of R and FR light). In contrast, Rosado et al. (2019) reported that silencing *SlPIF4* led to shorter hypocotyls at 30 °C but not at 25 °C under white light with a 12-h/12-h photoperiod. Additionally, Wang et al. (2020) did not observe any changes in hypocotyl length in *SlPIF4* mutants or overexpression lines grown under white light (12-h/12-h photoperiod) or in darkness. Recently, the same group found that different mutant alleles of *slpif4* are associated with shorter hypocotyls under continuous white light at both 25 and 32 °C (Zhu et al. 2024).

The study of functional conservation among PIFs in crop plants may yield new resources for plant breeding, given the center role of PIFs in the shade response, which is a major yield-limiting factor in many crop plants such as potato (*Solanum tuberosum*), peanut (*Arachis hypogaea*), wheat (*Triticum aestivum*), maize (*Zea mays*), sunflower (*Helianthus annuus*), soybean (*Glycine max*), and rice (*Oryza sativa*) (Donald 1968; Villalobos et al. 1994; Robson et al. 1996; Libenson et al. 2002; Boccalandro et al. 2003; Tang and Liesche 2017; Wille et al. 2017; Chen et al. 2020; Hu et al. 2020; Lyu et al. 2021; Postma et al. 2021; Wang et al. 2022; Golan et al. 2023; Lyu et al. 2023). The role of PIF in regulating SAS and thermomorphogenesis in crop plants and the conservation of PIF activity across species has been poorly investigated. This study addresses these questions using single and high-order mutations of the tomato *SlPIF4*, *SlPIF7a*, *SlPIF7b*, *SlPIF8a, and SlPIF8b*.

## Results

### SlPIF8a regulates tomato plants’ response to low R/FR

In recent work, we found that in Arabidopsis, the enhanced response to low R/FR at warm ambient temperatures is mediated by the PIF7 transcription factor, with minor contributions from PIF4 and PIF5 (Burko et al. 2022). Here, we explored whether this hierarchy is also conserved in tomato. To address this question, we mutated 5 members of the *SlPIF* family: *SlPIF4* (*Solyc07g043580*), *SlPIF7a* (*Solyc03g115540*), *SlPIF7b* (*Solyc06g069600*), *SlPIF8a* (*Solyc01g090790*), and *SlPIF8b* (*Solyc10g018510*), using CRISPR–Cas9 (Supplementary Fig. S1A). *SlPIF4* and *SlPIF7s* were selected as they were the most similar to the Arabidopsis *PIF4* (*AT2G43010*) and *PIF7* (*AT5G61270*), respectively (Rosado et al. 2016) (Supplementary Fig. S1B), the main regulators of hypocotyl elongation in Arabidopsis in response to low R/FR, high ambient temperature, and their interaction (Koini et al. 2009; Li et al. 2012; Fiorucci et al. 2020; Burko et al. 2022).

It appears that *SlPIF7s* may have undergone quantitative subfunctionalization, resulting in lowered expression of both *SlPIF7a* and *SlPIF7b* (Rosado et al. 2016), which suggests that they might have lost their role in the tomato response to low R/FR. Therefore, we decided to mutate *SlPIF8a* and *SlPIF8b* as well. Although Arabidopsis PIF8 (At-PIF8) does not play a role in the response to low R/FR, its high sequence similarity to At-PIF7 (Supplementary Fig. S1B) led us to speculate that SlPIF8s may play a role in promoting stem elongation in tomato. We initially mutate *SlPIF8a* along with *SlPIF4*, *SlPIF7a*, and *SlPIF7b* as *SlPIF8a* is highly expressed in the vegetative tissues of tomato plants, in contrast to *SlPIF8b* (Supplementary Fig. S2, A and B).

**Figure 1.**
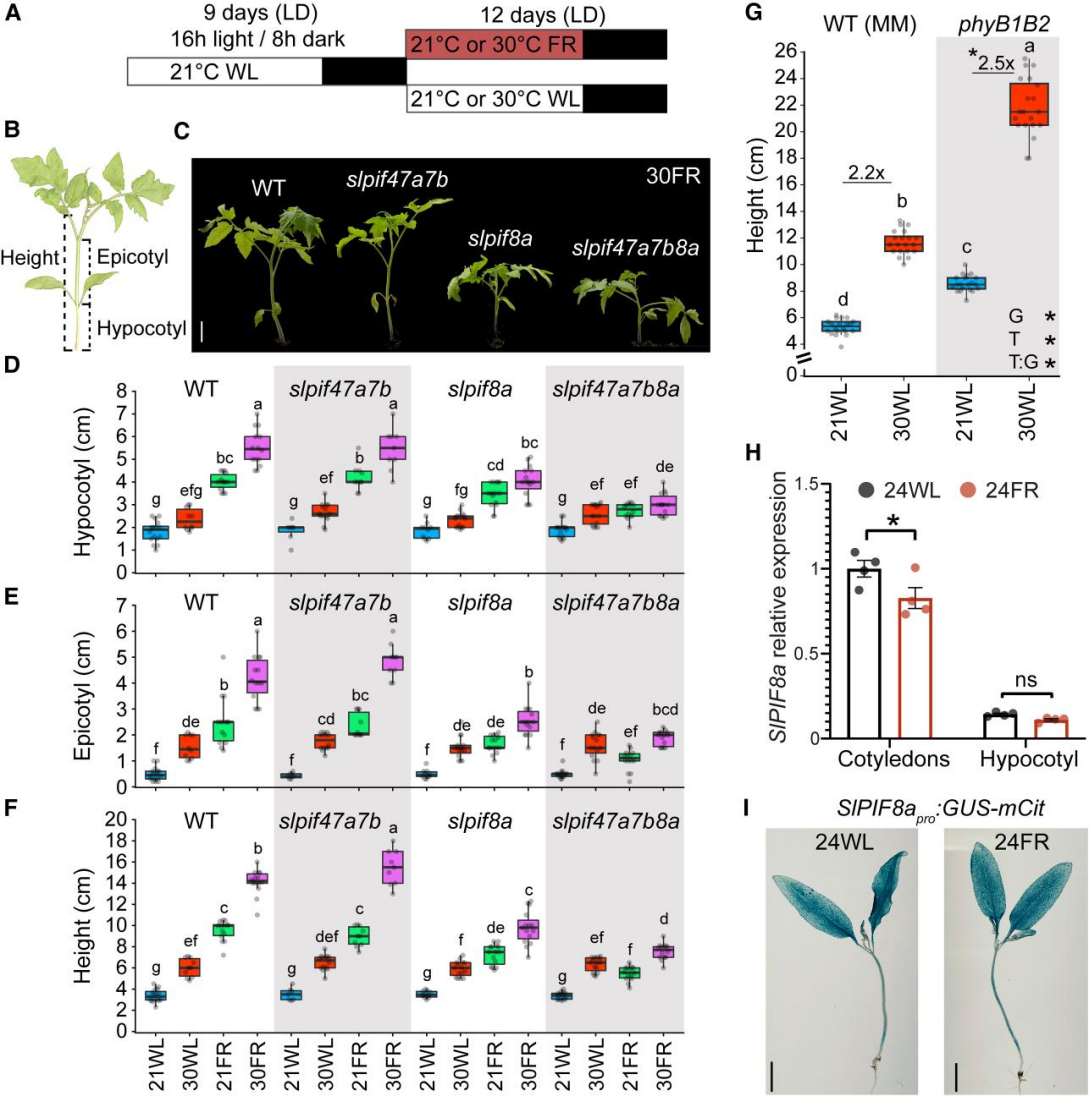
*SlPIF8a* plays a pivotal role in the response of tomato seedlings to low R/FR at 21 and 30 °C. **A)** Schematic illustration of the experimental design. Tomato seedlings were grown under LD conditions (16-h light/8-h dark) at 21 °C under white light (21WL, ∼200 *µ*mol m^−2^ s^−1^) for 9 days and then moved to LD + 21 °C + white light supplemented with FR light (21FR, ∼200 *µ*mol m^−2^ s^−1^, R/FR = 0.6), or LD + 30 °C + white light (30WL, ∼200 *µ*mol m^−2^ s^−1^), or LD + 30 °C + white light supplemented with FR light (30FR, ∼200 *µ*mol m^−2^ s^−1^, R/FR = 0.6), or kept at 21WL. **B)** A diagram of a tomato seedling in which the parts used for the measurements in this study are marked. **C)** Representative images of 21-day-old tomato seedlings of the indicated genotypes grown at LD 21WL for 9 days and then moved to LD 30FR for 12 more days. Images were digitally extracted for comparison. Scale bar = 3 cm (applicable to all images). WT, wild-type. **D)** Hypocotyl length, **E)** epicotyl length, and **F)** height of 21-day-old WT and *slpif* mutant seedlings grown as described in **A)**. Different letters denote statistical differences (*P* < 0.05) among samples, as assessed by 3-way ANOVA and Tukey's HSD. *n* > 7 seedlings per sample. **G)** Height of 21-day-old WT (cv. Moneymaker, MM) and *phyB1B2* mutant seedlings grown as described in **A)**. *n* > 18 seedlings per sample. The average fold change between 30 and 21 °C is presented. Different letters denote statistical differences (*P* < 0.05) among samples, as assessed using 2-way ANOVA and Tukey's HSD. An asterisk indicates a significant difference (*P* < 0.05), according to Student's *t*-test or ANOVA. T, temperature treatment; G, genotype; T:G, the interaction between temperature treatment and genotype. **H)** Relative expression of *SlPIF8a* in the cotyledons and hypocotyls of 9-day-old WT seedlings grown under LD conditions at 24 °C under white light (24WL, ∼200 *µ*mol m^−2^ s^−1^) and either kept under the same conditions or moved after 2 h of light to 24 °C supplemented with FR light (24FR, ∼200 *µ*mol m^−2^ s^−1^, R/FR = 0.6) for 4 h. Gene expression was assayed using RT-qPCR relative to the reference gene *EXPRESS* and normalized to the expression in the cotyledons at 24WL. The average values of 4 biological replicates per condition ± SE are shown. *ns*, not significant; *, significant differences at *P* < 0.05 according to Student's *t*-test. **I)** Images of GUS staining in the whole tomato seedlings carrying the *SlPIF8a_pro_:GUS-mCitrine* reporter gene. Plants were grown as described in **H)**, plus 24 h under the indicated condition. Scale bar = 1 cm. In **D)–G)**, boxes indicate the first and third quartiles, whiskers indicate the minimum and maximum values, the lines within the boxes indicate the median values, and the dots indicate individual data points.

As reported previously, exposure of wild-type (WT) tomato seedlings to low R/FR or warm ambient temperatures promotes the elongation of the hypocotyl and epicotyl and also increases the total plant height (Fig. 1, A–F and Supplementary Fig. S1C; Burko et al. 2022). Furthermore, the elongation response to low R/FR at warm temperatures was greater than that observed for each condition separately (Fig. 1, D–F and Supplementary Fig. S1C). To study the role of SlPIFs in promoting elongation in response to low R/FR and warm ambient temperatures, we generated a set of single, triple, and quadruple *slpif* mutants (Supplementary Fig. S1, A and B). We then grew those mutant plants under a long-day (LD, 16-h light/8-h dark) conditions and white light at 21 °C (21WL) until they were fully germinated (Supplementary Fig. S1D) and then either kept them under the same conditions or moved them to white light at 30 °C (30WL), low R/FR at 21 °C (21FR), or low R/FR at 30 °C (30FR, warm shade) (see Materials and methods and Fig. 1A). In response to low R/FR at 21 °C (21FR) or 30 °C (30FR), *slpif8a* and *slpif47a7b8a* (*slpifq*) mutants showed impaired elongation, whereas *slpif47a7b* (*slpift*) plants elongated similarly to the WT seedlings (Fig. 1, C–F and Supplementary Fig. S1E). These results suggest that, in tomato, SlPIF8a is a key regulator of the elongation response at 21FR and 30FR. Under our growth conditions, SlPIF4, SlPIF7a, and SlPIF7b each played a minor role that could be observed by the enhanced phenotype of *slpifq*, as compared to the *slpif8a* single mutant. To determine whether this is a unique scenario under LD conditions, we also exposed these mutants to low R/FR conditions under short-day (SD, 8:16) and day-neutral (12:12) light settings. While both *slpif8a* and *slpifq* mutants showed impaired elongation in response to low R/FR under day-neutral conditions, only *slpifq* exhibited reduced elongation under SD conditions (Supplementary Fig. 3, A and B). Surprisingly, all tested *slpif* mutants responded to warm temperatures similarly to WT seedlings under white-light (high R/FR) LD, SD, and day-neutral conditions (Fig. 1, D–F and Supplementary Figs. S1E and S3, C and D).

To elaborate on the role of SlPIF in tomato thermomorphogenesis, we tested whether the phyB-PIF module mediates this response by comparing the elongation response of the *phyB1B2* mutant with its corresponding wild type (cv. Moneymaker). Similar to the *slpif* mutants, we found that the relative response of the *phyB1B2* mutant is comparable to, or slightly stronger than the WT response to warm ambient temperatures under white-light LD, SD, and day-neutral conditions, as well as under LD at higher temperatures (Fig. 1G and Supplementary Fig. S3, E–G).

These results suggest that, in contrast to Arabidopsis, in tomato, SlPIF4, SlPIF7a, SlPIF7b, and SlPIF8a are not required for thermomorphogenesis-induced stem elongation under high R/FR (white-light) conditions across all the growth conditions that we tested. Notably, under low R/FR conditions, warm temperatures exacerbate the stem elongation response in an SlPIF-dependent manner (Fig. 1F, compare 21FR with 30FR). Therefore, we propose that while SlPIFs are not required to promote thermomorphogenesis-induced stem elongation under white-light conditions, under low R/FR conditions, when phyB repression of SlPIFs is lifted, their effect on elongation is enhanced by warm temperatures. This may also explain the slightly stronger response observed for *phyB1B2* under LD and SD conditions (Fig. 1G and Supplementary Fig. S3F).

Next, we explored whether the expression levels of *SlPIF8a* can explain its key role in the response to low R/FR. While *SlPIF8a* was expressed at higher levels in the cotyledons, as compared to the hypocotyl, its transcript level did not increase in response to low R/FR (Fig. 1H). Similarly, the expression levels of *SlPIF1a*, *SlPIF1b*, *SlPIF4*, *SlPIF7a*, and *SlPIF7b* did not increase in response to low R/FR (Supplementary Fig. S2C), as also demonstrated by Sun et al. (2020). Interestingly, *SlPIF3* expression in the cotyledons was upregulated in an SlPIF8a-dependent manner in response to low R/FR, suggesting that SlPIF3 may play a role in this response downstream of SlPIF8a (Supplementary Fig. S2C). In agreement with the RT-qPCR results, in response to low R/FR, no change was observed in the *SlPIF8a* expression domain in transgenic plants expressing *SlPIF8a_pro_:GUS-mCit* (Fig. 1I). We conclude that *SlPIF8a*'s response to low R/FR conditions at the transcript level cannot explain its dominant activity in regulating this response.

Since the Arabidopsis PIF8 has not been previously associated with the response to low R/FR and its role in regulating Arabidopsis light signaling is very subtle, as compared to the other PIF family members (Oh et al. 2020), we performed additional experiments to verify our findings. To that end, we studied the responses to low R/FR of a segregate plant; homozygote in *slpif4*, *slpif7a*, and *slpif7b*; and heterozygote in *slpif8a*. While seedlings with an active *SlPIF8a* allele elongated in a manner similar to that of the WT, seedlings homozygous for the *slpif8a* mutant allele failed to elongate (Supplementary Fig. S4A). In addition, we generated another allele of *slpif8a,* named *slpif8a^CR-2^* (Supplementary Fig. S4B), and found that the second allele perfectly mimicked the activity of *slpif8a^CR-1^* (the main allele used in this study; Supplementary Fig. S4C). Finally, we conducted a rescue experiment by expressing *SlPIF8a* in the *slpif8a* or *slpif47a7b8a* (*slpifq*) background. While the expression of *SlPIF8a* under its native promoter partially rescued *slpif8a* elongation, *SlPIF8a* driven by the constitutive *35S* promoter nearly fully rescued the elongation of *slpifq* plants in response to low R/FR (Supplementary Fig. S4, D and E).

Previous work has shown that SlPIF4 plays a role in the elongation response of tomato plants (Sun et al. 2020). However, in the current study, SlPIF4 did not contribute to the elongation response at 21 or 30 °C under low R/FR conditions. This could be due to the different tomato backgrounds used in the different studies. In the previous study, *slpif4* was generated in an indeterminate (ID) tomato background, carrying an active *SELF PRUNING* (*SP*) allele; whereas the current study was conducted in a determinant (D) background, that included the *sp* mutant allele, leading to early termination of the shoot apical meristem (Pnueli et al. 2001). Therefore, we introduced the *slpif4* and *slpif8a* mutants to the ID (*SP*/*SP*) background using crosses. Similar to the results observed in the D background, it was SlPIF8a and not SlPIF4 that played the major role in regulating the elongation response to low R/FR in the ID tomato plants (Supplementary Fig. S5A). Moreover, the *slpif4* mutants described by Sun et al. (2020) had no effect on tomato elongation under our growth conditions (Supplementary Fig. S5B).

Together, these results suggest that while SlPIF transcription factors mediate the response to low R/FR in tomato, they do not play a role in the plant's response to warm temperatures under white light under any of the examined growth conditions. Additionally, unlike in Arabidopsis, in which At-PIF7 plays the dominant role in mediating the response to low R/FR, in tomato, SlPIF8a mediates this response under LD and day-neutral conditions, but not under SD conditions. Notably, under different growth conditions and at various developmental stages, other SlPIFs may also play a role, as demonstrated by SlPIF4 during the early seedling stage (Rosado et al. 2019; Sun et al. 2020).

### Partially redundant role of SlPIFs

**Figure 2.**
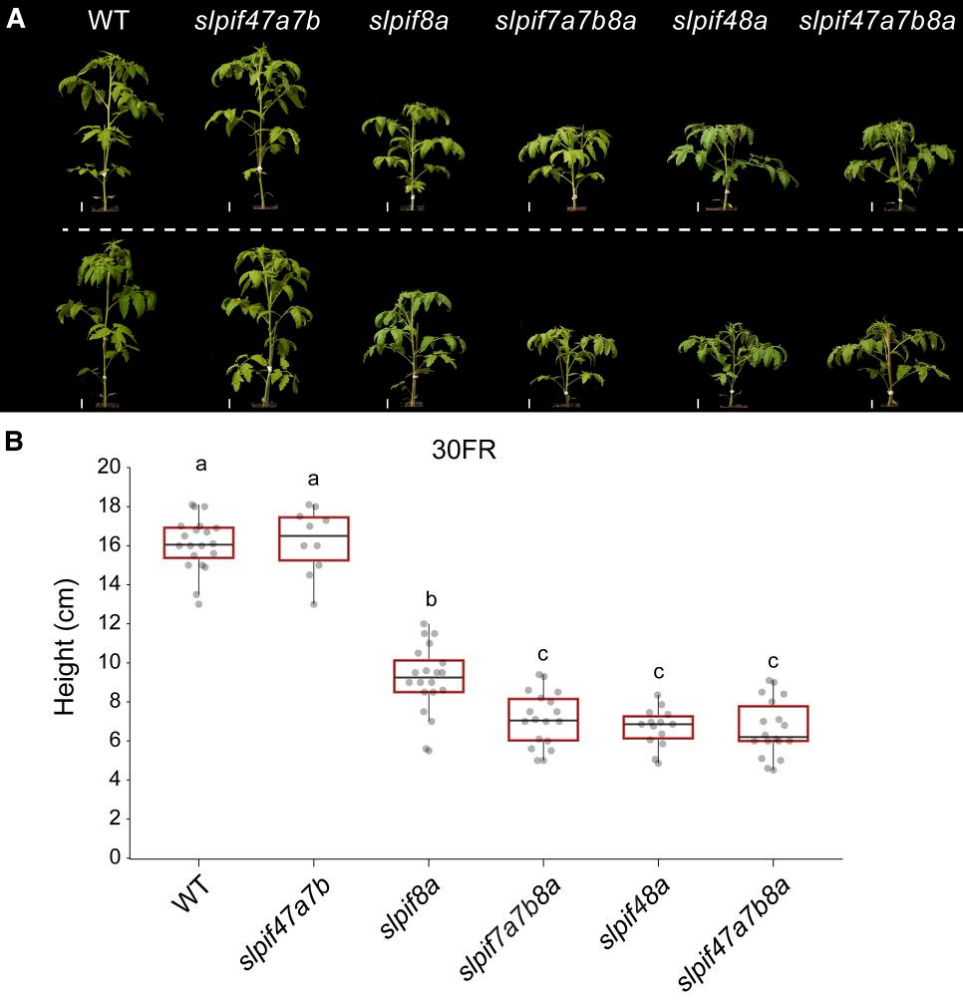
*SlPIF4* and *SlPIF7s* play minor roles in tomato plants’ response to low R/FR. **A)** Representative images of 32-day-old tomato seedlings of the indicated genotypes grown under LD conditions (16-h light/8-h dark) at 21 °C under white light (21WL, ∼200 *µ*mol m^−2^ s^−1^) for 9 days and then moved to LD + 30 °C + white light supplemented with FR light (30FR, ∼200 *µ*mol m^−2^ s^−1^, R/FR = 0.6) for 23 more days. Images were digitally extracted for comparison. Scale bar = 3 cm. WT, WT. **B)** Total height of 21-day-old WT, *slpif8a*, and the indicated *slpif* mutant plants. Seedlings were grown at 21WL for 9 days and then moved to 30FR for 12 more days. *n* > 10 seedlings per sample. Different letters denote statistical differences (*P* < 0.05) among samples as assessed by 1-way ANOVA and Tukey's HSD. Boxes indicate the first and third quartiles, whiskers indicate the minimum and maximum values, lines within the boxes indicate the median values, and the dots indicate the individual data points.

Our results place SlPIF8a at the center of the tomato response to low R/FR at 21 or 30 °C. However, we observed that *slpif47a7b8a* (*slpifq*) plants were slightly shorter than *slpif8a* plants under these conditions; whereas *slpif47a7b* did not affect plant height (Figs. 1F and 2). This suggests that SlPIF4, SlPIF7a, and SlPIF7b may play roles in the low R/FR response, which are masked by the dominant activity of SlPIF8a. To address this possibility and any possible redundancy among the SlPIFs, we examined the elongation of single, double, triple, and quadruple *slpif* mutants at 30FR. We found that while *slpif4* plants had WT-like responses to low R/FR, in the double mutant, *slpif48a* repressed the elongation response to the same extent as *slpifq* did (Fig. 2 and Supplementary Fig. S6A). Similarly, *slpif7a7b8a* plants mimicked the *slpifq* elongation phenotype, whereas *slpif7a7b* plants had WT-like responses and *slpif7a8a* and *slpif7b8a* plants had *slpif8a*-like responses (Fig. 2 and Supplementary Fig. S6, A and B).

In addition, since we identified SlPIF8a as the dominant SlPIF that regulates the low R/FR response, under our growth conditions, we examined whether *slpif8b*, the second tomato homolog of the Arabidopsis *PIF8*, plays a role in the low R/FR response. To that end, we generated *slpif8b* single and *slpif8a8b* double mutant plants. In agreement with the very low expression levels of *slpif8b* in the vegetative tissue (Supplementary Fig. S2A), the *slpif8b* mutant had a WT-like response and *slpif8a8b* plants had an *slpif8a*-like response, suggesting that, unlike SIPIF8a, SlPIF8b does not play a role in the response to low R/FR (Supplementary Fig. S6, A and C).

Altogether, we conclude that while SlPIF8a is sufficient to promote stem elongation in response to low R/FR (observed by the WT-like response of the *slpif47a7b* mutant), in the absence of SlPIF8a (*slpif8a* plants), SlPIF4, SlPIF7a, and SlPIF7b make relatively small, yet notable contributions to that response. In addition, our results suggest that SlPIF8b does not play a role in the response to low R/FR.

### SlPIF8a regulates gene expression and is regulated at the protein level similarly to Arabidopsis PIF7

**Figure 3.**
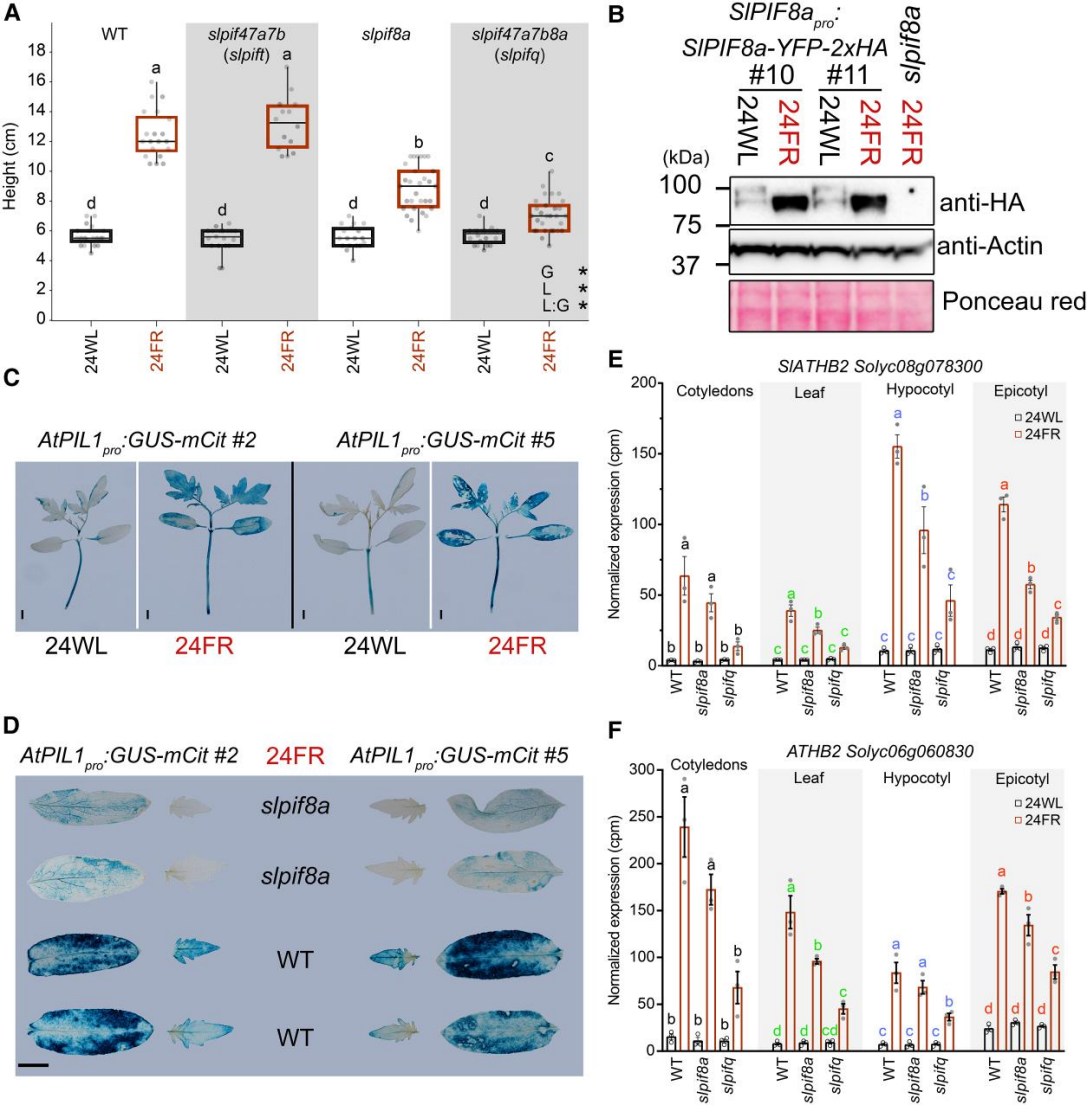
*SlPIF8a* is regulated by low R/FR and regulates gene expression much like Arabidopsis PIF7 does. **A)** Total height of 21-day-old WT plants and the indicated *slpif* mutants. The seedlings were grown under LD conditions (16-h light/8-h dark) at 24 °C under white light (24WL, ∼200 *µ*mol m^−2^ s^−1^) for 9 days and then moved to LD + 24 °C + white light supplemented with FR light (24FR, ∼200 *µ*mol m^−2^ s^−1^, R/FR = 0.6) for 14 more days. *n* > 14 seedlings per sample. Different letters denote statistical differences (*P* < 0.05) among samples, as assessed using 2-way ANOVA and Tukey's HSD. An asterisk indicates a significant difference (*P* < 0.05); L, light; G, genotype; L:G, the interaction between light condition and genotype. Boxes indicate the first and third quartiles, and whiskers indicate the minimum and maximum values. The lines within the boxes indicate the median values, and dots indicate the individual data points. **B)** Immunodetection of SlPIF8a-YFP-2xHA protein levels using anti-HA antibody. Total protein extract from the cotyledons of 2 lines was collected from 9-day-old *SlPIF8a_pro_:SlPIF8a-YFP-2xHA* (*slpif8a*) seedlings grown at 24WL, followed by 24 h at 24FR or 24WL. Anti-ACTIN blots and Ponceau red are shown below as loading controls. **C)** Images of GUS staining of 2 different tomato lines carrying the *AtPIL1_pro_:GUS-mCitrine* reporter gene. Plants were grown at 24WL for 12 days, followed by 12 h under the indicated condition. Images were digitally extracted for comparison. Scale bar = 0.7 cm. **D)** Images of GUS-stained WT or *slpif8a* cotyledons from plants carrying the *AtPIL1_pro_:GUS-mCitrine* reporter gene. The transgenic lines and growth conditions are the same as in **C)**. Images were digitally extracted for comparison. Scale bar = 0.7 cm (applicable to all images). Expression levels (derived from the RNA-seq data) of the tomato homologs of *At-ATHB2*: *Solyc08g078300*  **(E)** and *Solyc06g060830*  **(F)**. The expression levels in the indicated organs of WT, *slpif8a*, and *slpifq* plants, grown as described in Fig. 4B plus 6 h of 24FR or 24WL are shown. Data are presented as normalized cpm mapped reads. The average values of 3 biological replicates per condition ± SE are presented. Different letters denote statistical differences (*P* < 0.05) among samples as assessed by 1-way ANOVA and Tukey's HSD for each organ separately.

We identified that, in tomato, SlPIF8a plays a role in promoting the response to low R/FR, much like the role played by PIF7 in Arabidopsis. We, therefore, wondered whether SlPIF8a acts via target pathways that are similar to those used by PIF7 in Arabidopsis and whether these factors are regulated similarly at the protein level. To address these questions, we explored the effect of SlPIF8a on known At-PIF7 target genes and whether SlPIF8a protein modification during the response to low R/FR is similar to that observed for At-PIF7. Since we did not observe any effect of SlPIFs on warm temperature-mediated elongation of tomato seedlings grown under white light, and in order to work at the optimum tomato growth temperature, we performed all of the experiments discussed from this point forward at a temperature of 24 °C, focusing mainly on the low R/FR response. We first confirmed that the role of the SlPIFs in the response to low R/FR at 24 °C is similar to the 1 we observed at 21 and 30 °C (Figs. 1F and 3A). In Arabidopsis, At-PIF7 is phosphorylated in high R/FR and dephosphorylated in low R/FR light (Li et al. 2012; Willige et al. 2021). Therefore, we asked whether the modification of the SlPIF8a protein also changes in response to low R/FR. To that end, we generated tomato plants carrying the *SlPIF8a_pro_:SlPIF8a-YFP-2xHA* constructs and grew them under white light or exposed them to low R/FR light before extracting their protein. We found that while the SlPIF8a-*YFP-2xHA* protein under white light (high R/FR) ran as 2 bands, the protein under low R/FR ran as 1 band, implying a modification of the SlPIF8a protein in response to low R/FR light (Fig. 3B), similar to that observed for At-PIF7.

To test whether SlPIF8a can regulate At-PIF7 target genes, we compared the activity of the Arabidopsis *PHYTOCHROME-INTERACTING FACTOR 3-LIKE 1* (*PIL1*) promoter (*At-PIL1_pro_*), a well-studied promoter target of At-PIF7 (Li et al. 2012; Jiang et al. 2019), in WT and *slpif8a* mutant plants. To that end, we transformed tomato plants with *At-PIL1_pro_:GUS-mCit* and introduced the *slpif8a* mutation into 2 independent *At-PIL1_pro_:GUS-mCit* lines. We first confirmed that, as in Arabidopsis, *At-PIL1_pro_* is highly responsive to the low R/FR condition in tomato seedlings (Fig. 3C). Next, we found that the response to low R/FR of *At-PIL1_pro_:GUS-mCit* plants decreased when SlPIF8a was inactive (Fig. 3D).

To further explore the role of SlPIFs and, specifically, the role of SlPIF8a in regulating the responses of known At-PIF7 target genes to low R/FR (Willige et al. 2021), we compared the expression of the Arabidopsis *ARABIDOPSIS THALIANA HOMEOBOX2* (*ATHB2*) gene homologs *SlATHB2* (*Solyc08g078300*) and *ATHB2* (*Solyc06g060830*) among WT, *slpif8a*, and *slpifq* plants. To expand our question to the tissue level, we examined their expression levels in the cotyledons, hypocotyl, first leaf, and epicotyl. We observed that, as in Arabidopsis, in response to low R/FR, the levels of *SlATHB2* and *ATHB2* were significantly upregulated in all tissues (Fig. 3, E and F). In addition, the expression of *SlATHB2* and *ATHB2* was highly dependent on SlPIF activity in all of the tested organs, as observed by the expression gradient, with the highest levels of expression observed in the WT, lower levels in the *slpif8a* plants, and the lowest level of expression observed in *slpifq* plants (Fig. 3, E and F). These changes in the expression of *SlATHB2* and *ATHB2* are consistent with the stronger phenotype of *slpifq* plants, as compared to *slpif8a* plants, under these conditions (Fig. 3A).

Together, these results suggest that while PIFs mediate the response to low R/FR in both tomato and Arabidopsis, a different PIF plays the dominant role in mediating this response in each species: PIF7 in Arabidopsis and in tomato under our growth conditions it is SlPIF8a. Both PIFs are likely regulated in a similar manner at the protein level and can regulate similar genes.

### SlPIF8a regulates the response to low R/FR in all aerial organs

**Figure 4.**
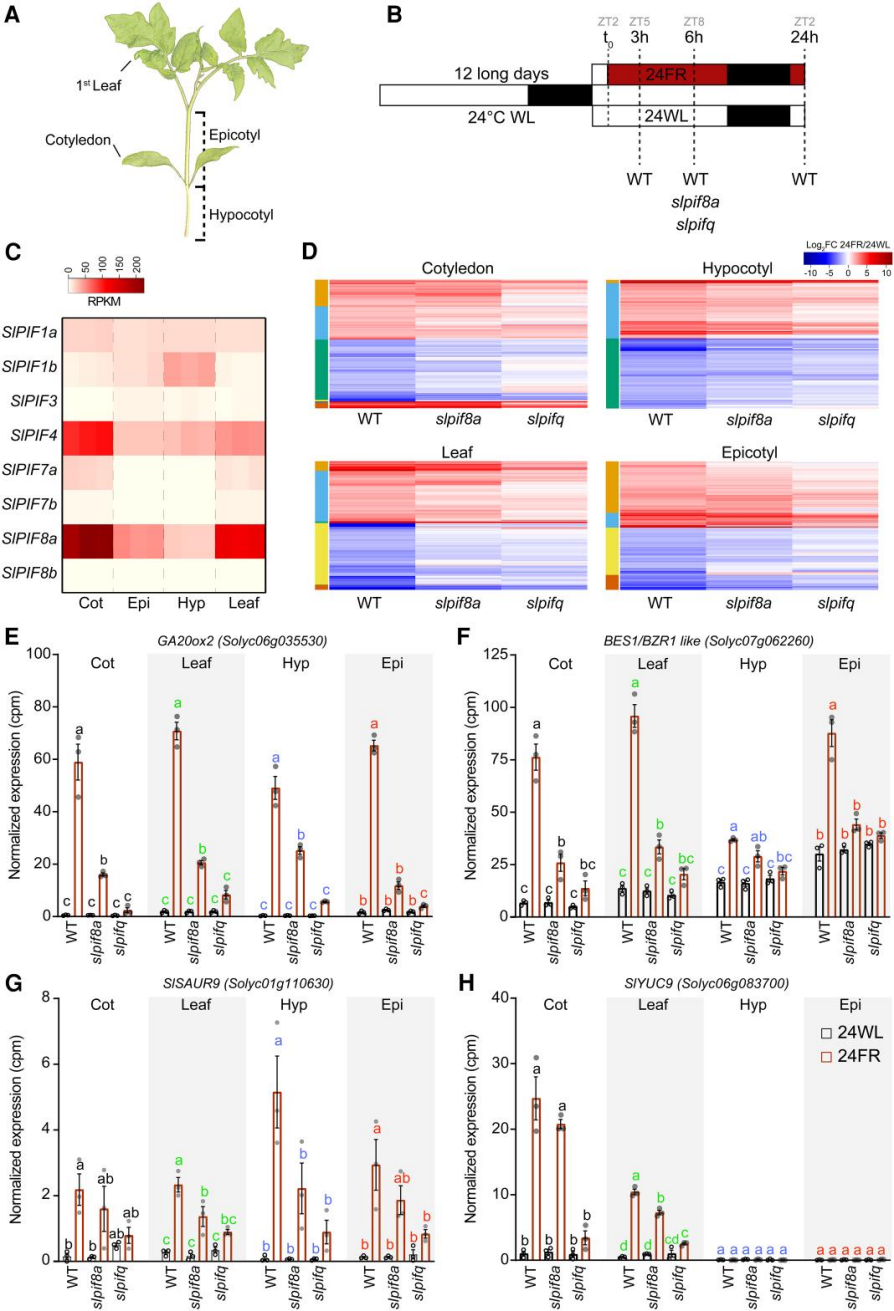
Tomato plants’ response to low R/FR is regulated by SlPIF4, SlPIF7a, SlPIF7b, and SlPIF8a. **A)** A diagram of a tomato seedling; the organs used for the RNA-Seq analysis are highlighted. **B)** A diagram of the experimental setup used for the RNA-Seq experiments. ZT, zeitgeber; *t*_0_, the time at which the FR light was turned on. WT, wild-type. 24WL, LD conditions (16-h light/8-h dark) at 24 °C under white light (∼200 *µ*mol m^−2^ s^−1^). 24FR, LD + 24 °C + white light supplemented with FR light (∼200 *µ*mol m^−2^ s^−1^, R/FR = 0.6). **C)** Tissue-specific expression levels (derived from the RNA-seq data) of tomato *SlPIF* genes in WT seedlings exposed to 5 h of white light (ZT5). The color scale represents the reads per kilobase per million mapped reads. **D)** Tissue-specific expression profiles of differentially expressed genes (FDR < 0.05 and FC > 1 or < −1) in WT, *slpif8a*, and *slpifq* plants at 6 h in 24FR relative to 24WL in WT seedlings. The color scale represents the log2 fold change relative to 24WL. The colors of the column on the left indicate the cluster numbers in each heatmap. Expression levels (derived from the RNA-Seq data) of tomato *GA20ox2* (*Solyc06g035530*, **E**), *BES1/BZR1-like* (*Solyc07g062260*, **F**), *SlSAUR9* (*Solyc01g110630*, **G**), and *SlYUC9* (*Solyc06g083700*, **H**). Data are presented as normalized cpm mapped reads. The average values of 3 biological replicates per condition ± SE are presented. Different letters denote statistical differences (*P* < 0.05) among samples, as assessed by 1-way ANOVA and Tukey's HSD for each organ separately. For **C)** and **D)**, see **Supplementary Data set 1**. In **D)–H)**, the expression levels in the indicated organs of WT, *slpif8a*, and *slpifq* plants grown as described in **B)**, and exposed to 6 h of 24FR or 24WL are presented. In **C**) and **E)–H)**, Cot, cotyledons; Leaf, first leaf; Hyp, hypocotyl; Epi, epicotyl.

While we found that SlPIF8a regulates gene expression similarly to At-PIF7, to expand our understanding of the role of SlPIFs, in general, and SlPIF8a, specifically, in mediating the tomato response to low R/FR, we performed RNA-seq. We compared the response to low R/FR among WT, *slpif8a*, and *slpifq* plants, in the hypocotyls, epicotyls, cotyledons, and first leaves of those plants (Fig. 4, A and B). Since it was recently shown that the expression levels of *PIFs* can determine their dominant activity (Kim et al. 2024), we first asked whether the expression level of *SlPIF8a* relative to all other *SlPIF*s can explain its dominant role in the response to low R/FR. We found that in young tomato seedlings grown in white light, *SlPIF8a* is expressed at substantially higher levels than *SlPIF7s* (in fact, at the highest level compared with all other *SlPIF*s), especially in the cotyledons and the first leaf, which are the organs that initiate the response (Fig. 4C and Supplementary Fig. S7A). This observation suggests that the dominant nature of *SlPIF8a* activity is at least partly due to its expression level in white light, before a plant is exposed to the low R/FR condition. Interestingly, in Arabidopsis, *At-PIF7*, which dominantly regulates the low R/FR response, is not expressed at a level higher than that of all the other *At-PIFs* (Supplementary Fig. S7B; Kohnen et al. 2016).

Next, we plotted all of the genes whose expression levels changed significantly in response to low R/FR, by organ. This revealed that *slpif8a* and *slpifq* suppressed the response to low R/FR in a gradual manner in all organs, reflecting their gradual effect on plant height at low R/FR (Figs. 3A and 4D and Supplementary Data Set 1). This suppression was evident among both genes that are upregulated by low R/FR and genes that are downregulated by low R/FR (Supplementary Fig. S7C). Among the genes found to be regulated by low R/FR and to be expressed in an SlPIFs-dependent manner were the cell wall-modifying genes *EXPANSIN A11* (EXPA11, Solyc04g081870) and *XYLOGLUCAN ENDOTRANSGLUCOSYLASE/HYDROLASE* (*XTH16/BR1*, *Solyc09g092520*) (Supplementary Fig. S8), which have also been shown to be regulated in tomato by a brassinosteroid (Koka et al. 2000). A similar pattern was also found for the hormone-related genes gibberellin (GA) biosynthetic gene *GA 20-oxidase 2* (*SlGA20ox2, Solyc06g035530*), GA-catabolic gene *GA 2-oxidases 9* (*SlGA2ox9, Solyc10g007570*), the brassinosteroid (BR) biosynthetic genes *PHYB ACTIVATION TAGGED SUPPRESSOR 1* (*BAS1, Solyc12g006860*) and BR signaling transcription factor *BES1/BZR1-like* (*Solyc07g062260*), the ethylene biosynthetic genes 1-AMINOCYCLOPROPANE-1-CARBOXYLATESYNTHASE 3 (*ACS3*, *Solyc02g091990*) and *1-aminocyclopropane-1-carboxylate oxidase 3* (*ACO3, Solyc07g049550*), the cytokinin (CK) catabolic gene *CK OXIDASE 5* (*CKX5*, *Solyc04g016430*), the abscisic acid biosynthetic (ABA) gene *NINE-CIS-EPOXYCAROTENOID DIOXYGENASE 3* (*NCED3*, *Solyc05g053530*), and the auxin-response gene *SlSAUR19* (*Solyc01g110630*) (Fig. 4, E–G and Supplementary Fig. S9). The auxin biosynthetic gene *SlYUC9* (*Solyc06g083700*) was expressed only in the cotyledons and the first leaf, and its expression was also dependent on SlPIFs (Fig. 4H). On a global scale, Gene Ontology (GO) analysis of the genes regulated by low R/FR and SlPIFs revealed common categories across all organs, including auxin response, GA response, and flavonoid biosynthesis (Supplementary Fig. 10).

Interestingly, a previous study found that the photosynthesis- and flavonoid-related genes are overrepresented (mainly downregulated) during tomato's response to low R/FR (Cagnola et al. 2012). However, our study only found the flavonoid-related genes to be overrepresented (Supplementary Fig. 10). This suggests that while SlPIFs regulate flavonoid-related genes, genes related to photosynthesis are regulated by low R/FR in an SlPIF-independent manner.

**Figure 5.**
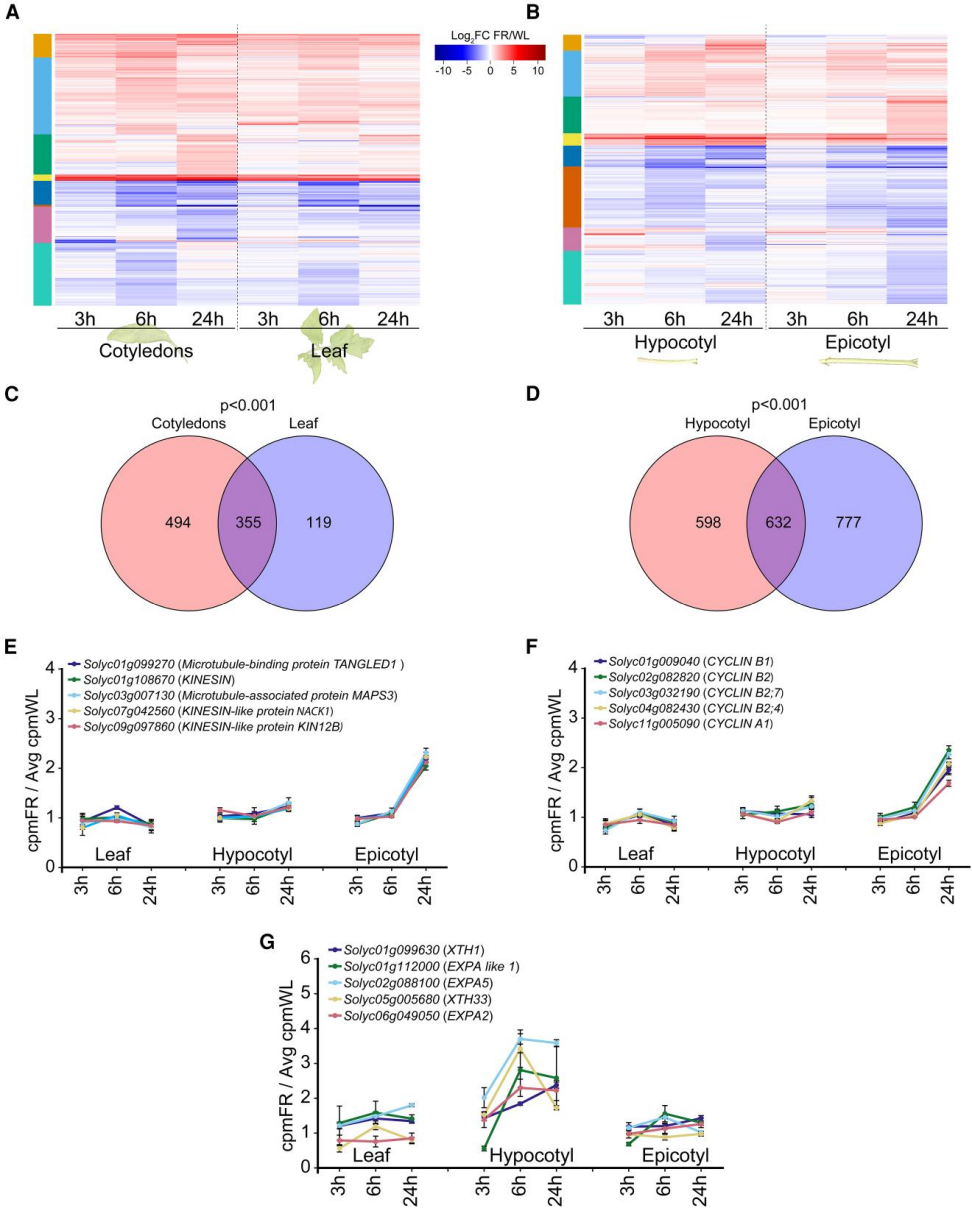
Response to low R/FR across the cotyledons and the first leaf and across the hypocotyl and the epicotyl. Expression profiles of genes differentially expressed in the cotyledons or the first leaf (24FR relative to 24WL, FC > 1 or < −1, FDR < 0.05) of WT plants **(A)** and in the hypocotyl or epicotyl **(B)** at at least 1 time point. For a list of genes in each organ and at each time point, see **Supplementary Data Set 2**. The color scale represents the log2 fold change relative to 24WL at the indicated time. The colors of the column on the left indicate the cluster numbers in each heatmap. Venn diagram comparing the genes expressed differentially at 24FR, at at least 1 time point, in the cotyledons as compared to the first leaf **(C)** and the hypocotyl as compared to the epicotyl **(D)**. The number of genes in each group is presented. Fisher's exact test was used to test for overrepresented gene overlap. For a list of genes in each group, see **Supplementary Data Set 2**. Relative expression at the indicated time point (cpm at 24FR/average cpm at 24WL), derived from RNA-seq data, for genes associated with **E)** cell cytokinesis, **F)** the cell cycle, and **G)** cell expansion in the first leaf, hypocotyl, and epicotyl. In **A)–G)** 24WL, LD conditions (16-h light/8-h dark) at 24 °C under white light (∼200 *µ*mol m^−2^ s^−1^) and 24FR, LD + 24 °C + white light supplemented with FR light (∼200 *µ*mol m^−2^ s^−1^, R/FR = 0.6).

To assess the similarity of the responses that occur in the cotyledons and the first leaf, as well as the similarity of the responses that occur in the hypocotyl and the epicotyl, we examined the genes that responded to low R/FR at least once and in at least one of these organs. This analysis revealed that the overall response in the cotyledons is very similar to the response in the first leaf (Fig. 5, A and C and Supplementary Data Set 2). At first glance, the overall response in the hypocotyl also seems similar to the response in the epicotyl (Fig. 5, B and D and Supplementary Data Set 2). However, upon comparing the GO categories for these 2 organs, we found that the epicotyl is exclusively enriched in categories related to the cell cycle and cell cytokinesis, whereas the hypocotyl shows enrichment in categories related to cell expansion, which were not found in the epicotyl (Supplementary Data Set 3). Therefore, we screened the differentially expressed genes in each organ, focusing on those associated with the cell cycle, cell cytokinesis, and cell expansion, and plotted their expression during the response to low R/FR in the hypocotyl, epicotyl, and first leaf, as a control (Fig. 5, E–G). We observed that *CYCLIN* genes, which regulate the cell cycle, and *KINESIN* genes, which are associated with cell division, were specifically upregulated in the epicotyl. In contrast, genes such as *EXPA2, EXPA5, XTH1,* and *XTH33*, which are associated with cell elongation, were specifically upregulated in the hypocotyl (Fig. 5, E–G). These observations suggest that the elongation of the tomato shoot in response to low R/FR occurs through 2 distinct mechanisms: cell elongation in the hypocotyl and cell division in the epicotyl.

Altogether, the results we present suggest that, as in Arabidopsis, in response to low R/FR, auxin is produced and accumulates in the tomato cotyledons and the leaves, from which it is most likely transported to the hypocotyl and the epicotyl, respectively, to promote elongation (Tao et al. 2008; Kozuka et al. 2010; Procko et al. 2014; Procko et al. 2016). Unlike Arabidopsis, in tomato, among all of the *SlPIF* genes, *SlPIF8a* was the most highly expressed in the cotyledons and first leaf. In addition, under the growth conditions used in this study, SlPIF8a is the main PIF regulating the response to low R/FR, while SlPIF4, SlPIF7a, and SlPIF7b play minor and redundant roles. In addition, we show that, like the hypocotyl and the epicotyl, the cotyledons and the first leaf exhibit overall similar responses to low R/FR, with the exception of cell division-associated genes, which are exclusively upregulated in the epicotyl, and cell elongation genes, which are exclusively upregulated in the hypocotyl.

## Discussion

Plants evolve to respond to their neighbors, to adapt to their local environment and better cope with population density. This response is initiated when the R/FR ratio drops below 1, triggering the photoconversion of phyB into its inactive form. PIF transcription factors have been shown to be the major factors that mediate the low R/FR response downstream of phyB. Using CRISPR–Cas9, we generated a set of mutants in the tomato *SlPIF* family, to reveal the roles of different SIPIFs in the response to low R/FR, study their activity hierarchy, and test their redundancy.

By studying the roles of tomato SlPIFs under low R/FR conditions and in the context of thermomorphogenesis responses, we revealed that, unlike in Arabidopsis, PIF8 plays a key role in regulating tomato's response to low R/FR. We identified SlPIF8a as a major player in mediating the low R/FR response in tomato under our growth conditions, while SlPIF4, SlPIF7a, and SlPIF7b play more subtle roles in this response. Surprisingly, under our white-light growth conditions, the phyB-PIF module in tomato is not required to promote the elongation response to warm ambient temperatures (Fig. 1, F and G and Supplementary Fig. S3). However, it is possible that, in tomato, other phytochromes and SlPIFs not examined in this study, such as homologs of phyA, PIF1, or PIF3, may also contribute to this response. Additionally, under different conditions, the phyB-PIF module may play a role in tomato response to warm temperatures. For example, Zhu et al. (2024) showed a loss of temperature responsiveness in *phyB1B2* and *slpif4* mutant seedlings, Rosado et al. (2019) showed a loss of temperature responsiveness in *SlPIF4*-silenced seedlings, and Sun et al. (2020) described shorter hypocotyls of *slpif4* mutant seedlings under white-light and shade conditions. The differences between our results and previous reports could be due to differences in growth conditions (such as light, humidity, and seedling stages), as well as the type of growing media (media plates versus soil) used in the different studies. Indeed, when we grew the *slpif4* line reported by Sun et al. (2020) under our growing conditions, we did not observe any change in hypocotyl length. Similarly, Wang et al. (2020) found no difference in tomato hypocotyl length in the presence of different alleles of *slpif4* mutant plants. Additionally, in the studies that observed phenotypic changes in *slpif4* mutants (Rosado et al. 2019; Sun et al. 2020; Zhu et al. 2024), seedlings were exposed to the tested conditions at a very early stage. Since tomato hypocotyl length is influenced by germination rate, we speculate that the differences in hypocotyl length observed in different studies may be attributed to variations in germination timing. In the current study, we minimized the impact of the differences in germination rate by transferring only fully germinated seedlings (9 days old) to the tested growing conditions. Moreover, we measured epicotyl length, total plant height, and hypocotyl length. Therefore, while *SlPIF4* does play a role in regulating hypocotyl elongation in very young seedlings and under specific growth conditions, the results presented in this study suggest that this is not the case in slightly more mature plants. It is worth noting that in the *slpif8a* background, *SlPIF4* played a minor role in mediating the low R/FR response (Fig. 2).

### Evolution of species-specific PIF activity

Eight PIF-encoding loci have been identified in the *S. lycopersicum* genome: *SlPIF1a*, *SlPIF1b*, *SlPIF3*, *SlPIF4*, *SlPIF7a*, *SlPIF7b*, *SlPIF8a*, and *SlPIF8b*. It has been suggested that *SlPIF1*, *SlPIF7,* and *SlPIF8* duplications may have occurred during the *Solanum* lineage polyploidization event 71 (± 19.4) MYA, prior to the divergence of tomato and potato species (Rosado et al. 2016). That same study suggested that, in tomato, *SlPIF7a* and *SlPIF7b* may have gone through quantitative subfunctionalization that reduced their expression levels (Rosado et al. 2016). The substantially lower expression of *SlPIF7s* relative to *SlPIF8a*, the dominant function of SlPIF8a, and the minor role of SlPIF7s in the response to low R/FR support the hypothesis that tomato SlPIF7s lost their functionality or acquired a new function. Similar to what we observed in tomato, *CaPIF8* has been shown to be the most highly expressed of all of the *CaPIFs* in pepper (*Capsicum annuum*) leaves and may potentially regulate plant height (Yang et al. 2021). This suggests that the loss of PIF7 activity may have occurred early in the evolution of the *Solanaceae*. In addition, it has been suggested that in *Populus* trees, which lack an *At-PIF7* homolog, PIF8 may regulate plant height, as in tomato (Ding et al. 2021). Yet, it is still not known what evolutionary events led to the specific activities of PIF7 in Arabidopsis and SlPIF8 in tomato.

It will be necessary to study the role of PIFs in mediating the shade response in additional species, to better understand the evolutionary process that has led to their species-specific activity. Overexpression of different *PIFs* in Arabidopsis has been shown to promote elongation even in the light, while their mutants suggest that the different PIFs play specific roles within a particular process (Li et al. 2012; Oh et al. 2020; Kim et al. 2024). Therefore, it appears that their roles are determined through a delicate regulatory system that includes their expression levels, activity (stability and binding to target genes), and interaction with other proteins (Kim et al. 2024). Our finding that SlPIF8a can regulate the *At-PIL1* promoter together with the observation that, in Arabidopsis, both At-PIF7 and At-PIF8 bind to the *PIL1* promoter, but only At-PIF7 regulates the response to low R/FR (Li et al. 2012; Oh et al. 2020), argues against the possibility that the binding targets may be a major factor in determining the specificity of At-PIF7 and SlPIF8a. However, LONG HYPOCOTYL IN FAR-RED (HFR1), which interacts with and represses the activity of At-PIF7 in Arabidopsis (Hornitschek et al. 2009; Paulisic et al. 2021), is not present in the tomato genome, suggesting a different protein–protein interaction in Arabidopsis as compared to tomato, which might determine the species-specific function of the PIFs. In line with this idea, Paulisic et al. (2021) compared HFR1-PIF in Arabidopsis and *Cardamine hirsuta* and found enhanced total HFR1 activity to be accompanied by attenuated PIF activity in *C. hirsuta*, which reduced PIF activity and attenuated other PIF-mediated responses.

Interestingly, the tomato SlPIF8a and SlPIF7s did not acquire or conserve (respectively) the role of At-PIF7 in regulating the Arabidopsis thermomorphogenesis response under white light (Chung et al. 2020; Fiorucci et al. 2020; Burko et al. 2022). In addition, Rosado et al. (2016) suggested an evolutionarily conserved function of the PIF4 clade based on coincident expression patterns between tomato *SlPIF4* and Arabidopsis *PIF4* and *PIF5*. However, our finding that SlPIF4 does not play a role in the warm-temperature response and that the *slpif4* single mutant exhibited no visible phenotype in response to low R/FR suggests that their activity across these 2 plant species is not conserved.

Since in our white-light growth conditions SlPIF4, SlPIF7s, and SlPIF8a do not play any roles in the elongation response under warm temperatures, it could be that other SlPIF family members have acquired this function, especially in the light of the substitution between the activity of At-PIF7 and SlPIF8a. Alternatively, as tomato's natural growth temperatures are warmer than those of Arabidopsis, it might be that, in tomato, the PIFs were lost or did not acquire a role in mediating stem elongation in response to warm temperatures. While we did observe an elongation response to warm temperatures under white light and warm temperatures enhanced the low R/FR response, future studies might aim to identify the mechanism that mediates this response, which is likely independent of PIF.

### Organ-specific response to low R/FR

Studying the response to low R/FR in a caulescent plant such as tomato enabled us to compare the response to low R/FR of the hypocotyl with that of the epicotyl and the response of the cotyledons with that of the first leaf. In Arabidopsis, the response to low R/FR is elicited by both local and distal auxin signals. The distal auxin response depends on the upregulation of auxin biosynthetic genes in the cotyledons; whereas the local response can be observed in both hypocotyl and cotyledons at early time points (Kohnen et al. 2016).

Our results suggest that the tomato response also follows a very similar pattern. As shown previously for several plant species (Procko et al. 2014; Kohnen et al. 2016; Procko et al. 2016), we observed the upregulation of auxin biosynthetic genes specifically in the cotyledons and the first leaf, as well as the upregulation of auxin-, BR-, and GA-response genes in all of the examined organs (Fig. 4, E–H, Supplementary Figs. S8 and S9, and Supplementary Data Set 4). However, our time-course resolution was not high enough to allow us to determine the order of these events. In addition to auxin, genes involved in the biosynthesis of GA, BR, ethylene, CK, and ABA have been shown to be regulated by low R/FR in Arabidopsis (Hisamatsu et al. 2005; Kohnen et al. 2016; Hayes et al. 2019; Liu et al. 2021). Similarly, in tomato, we found that the expression of these genes was induced by low R/FR (Supplementary Fig. S9). In addition, and as in Arabidopsis, in which the genes’ responses to low R/FR in the hypocotyl, petiole, and lamina are PIF-dependent (Li et al. 2012; Michaud et al. 2017; Pantazopoulou et al. 2017; Burko et al. 2022; Oskam et al. 2024), the expression of many genes involved in the response of tomato plants to low R/FR was also found to be SlPIFs-dependent in all of the examined organs (Fig. 4, D–H, Supplementary Figs. S8 to S10, and Supplementary Data Set 5). Notably, their expression was not fully suppressed in the *slpifq* mutant (Figs. 3, E and F and 4, D–H), which was reflected by the slight elongation of the stem in *slpifq* mutant (Figs. 1, D–F and 3A). In contrast, in Arabidopsis, the genes induced by low R/FR are almost entirely abolished in the *pif457* mutant (Ince et al. 2022; Pastor-Andreu et al. 2024), consequently leading to the full suppression of the hypocotyl elongation. These observations suggest that in tomatoes, other SlPIFs may play a role, though minor, in regulating the response to low R/FR.

In addition, in Arabidopsis, the expression of a member of the GA-biosynthetic gene family, *At-GA20ox*, was low R/FR-induced in hypocotyls, and the expression of the GA-catabolic gene *At-GA2ox8* was low R/FR-induced in the cotyledons (Leivar and Quail 2011; Kohnen et al. 2016). However, in tomato, *SlGA20ox* and *SlGA2ox* were upregulated or downregulated, respectively, by low R/FR in all of the examined organs, in an SlPIFs-dependent manner (Fig. 4E and Supplementary Fig. S9F). In support of our observation that low R/FR regulates auxin, GA, and BR in a SlPIF-dependent manner, a recent paper demonstrated the importance of these hormones in tomato internode elongation and in response to low R/FR (Li et al. 2024).

All together and in contrast to Arabidopsis, in which the expression of low R/FR-response genes follows an opposite pattern in the hypocotyls as compared to the cotyledons, in tomato, many genes are expressed in a similar pattern in the hypocotyl, cotyledons, first leaf, and epicotyl. These observations suggest different responses to low R/FR in Arabidopsis, a rosette plant, and tomato, a caulescent plant with a very different growth habit. Our observation that genes associated with cell elongation were mainly upregulated in the hypocotyl, while genes associated with cell division were exclusively upregulated in the epicotyl in response to low R/FR emphasizes the differences between the Arabidopsis and tomato elongation responses (Fig. 5, E–G). Additionally, these observations suggest that the elongation of the tomato shoot in response to low R/FR, and potentially other environmental signals as well, proceeds via 2 distinct mechanisms: cell elongation in the hypocotyl, as described for the Arabidopsis and *Brassica rapa* hypocotyl (Gendreau et al. 1997; Procko et al. 2014; Das et al. 2016; Pastor-Andreu et al. 2024), and cell division in the epicotyl. This hypothesis will need to be addressed in future research, along with the role of SlPIFs in regulating both elongation mechanisms in an organ-dependent manner.

### Relevance for agriculture

Maximizing crop yield and quality while minimizing inputs and negative environmental impact is essential for sustainable food production. Unraveling the intricate molecular mechanisms underlying plant growth and development is necessary to achieve these goals. The multifaceted roles of PIF transcription factors in regulating plant growth, development, and stress responses highlight their potential importance for crop improvement programs. PIFs have been associated with fruit ripening, carotenoid levels, flowering time, and fruit size in tomato; with grain size and yield in rice; and with anthocyanin accumulation in apple (*Malus domestica*) (Llorente et al. 2016; Gao et al. 2018; Gramegna et al. 2019; Ji et al. 2019; Rosado et al. 2019; Wang et al. 2020; Pan et al. 2021; Simon-Moya et al. 2021; Liu et al. 2022). In addition, *TaPIF3* has been shown to regulate stem elongation and head length in wheat and *GhPIF4a* has been shown to be associated with cotton (*Gossypium hirsutum*) flowering time in a temperature-dependent manner (Sibbett 2018; Liu et al. 2023).

The harnessing of the regulatory functions of PIFs and their species-specific activity offers promising avenues for enhancing crop productivity, resilience, and sustainability in the face of global food-security challenges. The development of crops with altered PIF expression levels through genetic engineering or classical breeding approaches could mitigate yield losses associated with shade avoidance, improving crop productivity under high-density planting regimes. Our identification of SlPIF8a as a major factor controlling this response in tomato positions it as a feasible target for such manipulation.

PIFs also play roles in plant responses to other abiotic and biotic stresses. For example, PIF8 may be involved in cold and salt stress in pepper, in powdery mildew resistance in melon (*Cucumis melo*), and in seasonal growth in *Populus* (Ding et al. 2021; Yang et al. 2021; Wang et al. 2023). In addition, suppression of shade-induced upward leaf movement in the Arabidopsis *pif7* mutant has been shown to increase its ability to compete with invasive plants (Pantazopoulou et al. 2021). These observations emphasize the importance of a broad view of how an impairment in the response to low R/FR could affect a plant's ability to cope with a dynamic environment. Further research into the molecular mechanisms underlying PIF-mediated processes and their interactions with environmental factors should facilitate the development of innovative crop improvement strategies for a changing world.

## Materials and methods

### DNA constructs and plant transformation

The multisite Gateway system (Invitrogen) was used for the reporter *SlPIF8a_pro_:GUS-mCit* and expression lines *SlPIF8a_pro_:SlPIF8a-YFP-2xHA* and *35S_pro_:SlPIF8a*. To clone the *SlPIF8a* promoter, a 1,766-bp fragment upstream of the *SlPIF8a* start codon was amplified by PCR from M82 genomic DNA and inserted into pDONR221 P4-P1r. To clone *SlPIF8a,* its coding sequence was amplified by PCR from M82 cDNA and inserted into pDONR221 P1 to P2. *SlPIF8a_pro_:GUS-mCitrine* was generated by recombining *SlPIF8a* promoter (in pDONR221 P4 to P1r) with *GUS* (in pDONR221 P1 to P2) and *mCitrine* (in pDONR221 P2r to P3) (Procko et al. 2016) into the destination vector pK7m34GW.

*SlPIF8a_pro_:SlPIF8a-YFP-2xHA* was generated by recombining the *SlPIF8a* promoter (in pDONR221 P4 to P1r) with the *SlPIF8a* coding sequence (in pDONR221 P1 to P2) and the *YFP-2xHA* tag (HA-YFP-HA in pDONR221 P2r to P3) (Burger et al. 2017) in the destination vector pK7m34GW. *35S_pro_:SlPIF8a* was generated by recombining the 2*×35S* promoter (in pDONR221 P4 to P1r) with the *SlPIF8a* coding sequence (in pDONR221 P1 to P2) and the linker sequence “aagctcggatcccgggtacctcgg” (in pDONR221 P2r to P3) in the destination vector pK7m34GW. The Arabidopsis (*Arabidopsis thaliana*) *PIL1p:GUS-mCit* cloning was described in Burko et al. (2022).

For the CRISPR–Cas9 lines, the Golden Gate assembly method was used (Werner et al. 2012; Brooks et al. 2014) to generate a binary vector containing a CRISPR cassette with *35S_pro_:Cas9* and either 2 or 8 guide RNAs (gRNAs). To check the specificity of each gRNA target site, including the PAM site (NGG), BLAST analyses were performed against the tomato genome (SL2.50) using CRISPR-P (Liu et al. 2017). For the CRISPR/Cas9 construct targeting the *SlPIF*s coding region, 2 gRNA target sites (with a minimum of 4 mismatches to off-target sites) were selected for each gene (Supplementary Figs. S1A, S4B, and S6C). To produce each of the gRNAs, a PCR was carried out with a primer containing the gRNA sequence and a universal primer (Supplementary Table S1), using the plasmid pICH86966:: AtU6p::gRNA_PDS (Addgene plasmid 46966) as a template. To generate the *slpifq-*quadruple mutant plant (carrying the mutant alleles *slpif4^CR-1^*, *slpif7a^CR-1^*, *slpif7b^CR-1^*, and *slpif8a^CR-1^*), each gRNA was first assembled into Level 1 vectors together with pICSL01009_U6pro (Addgene plasmid 46968) using BsaI enzyme. gRNA1 and gRNA8 were cloned into pICH47751 (Addgene plasmid 48002), gRNA2 was cloned into pICH47761 (Addgene plasmid 48003), gRNA3 was cloned into pICH47772 (48004), gRNA4 was cloned into pICH47781 (Addgene plasmid 48005), gRNA5 was cloned into pICH47791 (Addgene plasmid 48006), gRNA6 was cloned into pICH47732 (Addgene plasmid 48000), and gRNA7 was cloned into pICH47742 (Addgene plasmid 48001). All 8 gRNAs were then assembled into 2 intermediate Level M vectors: pAGM8055 (Addgene plasmid 48039) with pICH50927 (Addgene plasmid 48049) or pAGM8093 (Addgene plasmid 48043) with pICH50892 (Addgene plasmid 48046), using BpiI enzyme. The Level 1 vectors pICH47732-NOSpro::NPTII (Addgene plasmid 51144), pICH47742-35S*_pro_*:Cas9 (Addgene plasmid 49771), pAGM8055 (Addgene plasmid 48039), pAGM8093 (Addgene plasmid 48043), and pICH41766 (Addgene plasmid 48018) were then assembled into the binary Level 2 vector pAGM4723 (Addgene plasmid 48015), using BpiI and BsaI enzymes to obtain the final binary plasmids containing all 8 gRNAs. To generate *slpif8a^CR-3^ slpif8b^CR-2^* double, *slpif8b^CR-1^* and *slpif8a^CR-2^* single alleles (Supplementary Figs. S4B and S6C), their gRNAs were cloned into Level 1 vectors pICH47751 (gRNA1), pICH47761 (gRNA2), pICH47772 (gRNA3), and pICH47781 (gRNA4). Level 1 constructs pICH47732-NOSpro::NPTII, pICH47742-35S*_pro_*:Cas9, pICH41822 end-link (Addgene plasmid 48021), and the Level 1 vectors containing the gRNAs were then assembled in the binary Level 2 vector pAGM4723 using BpiI enzyme.

*Agrobacterium tumefaciens* GV3101 and the cotyledon transformation method (McCormick 1997) were used to transform *SlPIF8a_pro_:SlPIF8a-YFP-2xHA* and *35S_pro_:SlPIF8a* into *slpif8a* and *slpifq*, respectively. All other constructs were transformed into M82 plants. Segregation analysis of antibiotic resistance was used to isolate single-insertion homozygous transgenic lines and specific primers for the Cas9 sequence were used to isolate *slpif* mutants, free of the CRISPR cassette (Supplementary Table S1).

### Genetic material

All tomato (*S. lycopersicum*) plants generated during this work had the M82 (*sp*) background. Unless otherwise specified, the *slpif* mutant alleles used in this study were: *slpif4^CR-1^*, *slpif7a^CR-1^*, *slpif7b^CR-1^*, and *slpif8a^CR-1^*. T_0_ plants carrying the 8-gRNA cassette were crossed with M82 (*sp*) to generate the *slpif47a7b8a (slpifq)*, *slpif47a7b (slpift), slpif7a7b8a*, *slpif7a7b*, *slpif48a*, *slpi7a8a*, *slpif7b8a*, *slpif4*, and *slpif8a* mutants free of CAS9. All assays were conducted using T3 or T4 seedlings. To generate the *slpif4* and *slpif8a* single mutant in the ID background, the *slpifq* plant was crossed with an M82 ID (*SP/SP*) plant. To get the *PIL1p:GUS-mCit* in the *slpif8a* background, 2 different lines of *PIL1p:GUS-mCit* were crossed with a *slpif8a* mutant plant and the *slpif8a* homozygote plants were identified using PCR (primers listed in Supplementary Table S1). The *slpif4* mutant in Ailsa Craig's background was described previously (Sun et al. 2020). “Moneymaker” (accession LA2706) and *phyB1B2* (accession LA4364) seed were obtained from the UCDavis/C.M. Rick Tomato Genetics Resource Center (TGRC), University of California, Davis.

### Growth conditions and stem measurements

Tomato plants were germinated in standard growing soil within a growth chamber equipped with LED white light, under LD (16-h light/8-h dark) conditions. Exceptions are noted in Supplementary Fig. S3, A–F, where fluorescent light was used (see light spectrum in Supplementary Fig. S11, B and C). The plants were grown at 21 °C (∼200 *µ*mol m^−2^ s^−1^, Supplementary Fig. S11A) for 9 to 12 days and then either kept under the same conditions (**21WL**) or transferred to LD at 30 °C (**30WL**, ∼200 *µ*mol m^−2^ s^−1^, R/FR = 14) or LD at 21 °C supplemented with FR light during the day-time (**21FR**, ∼200 *µ*mol m^−2^ s^−1^, R/FR = 0.6, Supplementary Fig. S11A) or LD at 30 °C supplemented with FR during the day-time (**30FR**, ∼200 *µ*mol m^−2^ s^−1^, R/FR = 0.6), for the period of time described in each figure legend. All temperatures were kept constant during the day and the night. The same settings were used for the 24 °C experiments, except that the plants germinated and grew at a constant 24 °C. The hypocotyls, epicotyls, and total height were measured with a ruler at the end of each experiment.

### Immunoblot analysis

Western blotting was performed on protein extracts from the cotyledons of 4 *SlPIF8a_pro_:SlPIF8a-YFP-2xHA* seedlings. The tissue was homogenized and boiled in 4X loading buffer with 3.6% β-mercaptoethanol (NuPAGE LDS sample buffer—ThermoFisher NP008) for 5 min and then diluted 1:1 with water. Extracts were run on a Bis-tris 4 to 12% acrylamide gradient gel (Invitrogen, Carlsbad, CA, USA) and then transferred to a 0.4-*µ*m nitrocellulose membrane by wet transfer. The membrane was cut in half to detect both the SlPIF8a-YFP-2xHA and the actin. The primary antibody anti-HA-HRP (1:2,000, catalog no. 12013819001, Roche) was used to detect SlPIF8a-YFP*-*2xHA. Antiactin (1:20,000, catalog no. A0480, Sigma) and the secondary goat antimouse IgG (H + L)-HRP conjugate (1:20,000, catalog no. 1706516, Bio-Rad) were used to detect the actin. The Sapphire Biomolecular Imager (Azure Biosystems) was used for imaging.

### Phylogenetic analysis

For the phylogenetic analysis, MEGA software 10.0.13 (Tamura et al. 2021) was used with the neighbor-joining method and Poisson model, pairwise deletion, and bootstrap (1,000 replicates; random seed) parameters. See Supplementary Files 1 and 2 for alignment and tree files.

### GUS staining

GUS (β-glucuronidase) staining was performed as described previously (Ori et al. 2000). Briefly, plant tissue was vacuum-infiltrated in staining solution (25 mm phosphate buffer, pH 7, 0.25% Triton X-100, 1.25 mm potassium ferricyanide, 1.25 mm potassium ferrocyanide, 0.25 mm EDTA, 1 mg/ml 5-bromo-4-chloro-3-indolyl β-D-glucuronide X-Gluc) and then incubated overnight at 37 °C. The tissue was then cleared in 95% ethanol and gradually brought to 50% ethanol with sequential washes and then to 50% glycerol.

### Reverse transcription quantitative PCR

For reverse transcription quantitative PCR (RT-qPCR), RNA was extracted from the hypocotyl, epicotyl, cotyledons, or first leaf of 4 tomato seedlings per biological replicate using the RNeasy Micro Kit (Qiagen). cDNA synthesis was performed using the Maxima first-strand cDNA synthesis kit (Thermo Fisher Scientific) with 1 *µ*g of RNA. RT-qPCR analysis was carried out using CFX384 Real-Time PCR Detection System (Bio-Rad), with Premix Ex Taq II (TaKaRa, #RR820A). Levels of mRNA were calculated relative to *EXPRESS* (*Solyc07g025390*) as an internal control. The primers used for the RT-qPCR analysis are listed in Supplementary Table S1.

### Statistical analyses

The statistical significance of differences was evaluated using Student's *t*-test, where “*” indicates significant differences at *P* < 0.05, or by 1-way, 2-way, or 3-way ANOVA followed by Tukey's HSD multiple comparison test which was performed using R (Supplementary Data Set 6). Different letters indicate significant differences at *P* < 0.05.

### RNA-seq experiments and analysis

Three biological replicates of WT, *slpif8a*, and *slpifq* plants were collected and snap-frozen in liquid nitrogen (Fig. 4, A and B). Total RNA was isolated from the hypocotyls, cotyledons, epicotyls, and first leaves of 4 tomato seedlings per biological replicate, using the RNeasy Micro Kit (Qiagen). Stranded mRNA-seq libraries were prepared using the Illumina TruSeq Stranded mRNA Library Prep Kit according to the manufacturer's instructions. Libraries were sequenced with single-end, 100-bp reads in the Illumina NovaSeq6000 System. Raw reads (two fastq files for each sample) were aligned to the SL4.0 genome using STAR (v2.7.9a) (Dobin et al. 2013) with default parameters except --alignIntronMax 2000 and --outFilterMismatchNmax 10. Mapped reads were counted by HTSeq (v0.13.5) with default parameters except “-m intersection-strict -s reverse” (Anders et al. 2015), and differential expression was determined using edgeR (v3.40.2) (Robinson et al. 2010; Chen et al. 2016). The WT (time-course) analysis and the comparison between WT, *slpif8a*, and *slpifq* were conducted separately. Genes with low counts per million (cpm) values were filtered out, using the edgeR function [rowSums(cpm(counts) > 5) ≥ 3] for differential expression analysis. A design matrix was set up in which each parameter (light, tissue, and time point for the WT time course, and light, tissue, and genotypes for the mutant analysis) was 1 group, and a generalized linear model was fitted to the read counts with this design matrix. For differential gene expression, contrasts were set up between 24FR and 24WL at each time point and for each tissue, as well as between genotypes for each condition and tissue.

Gene expression (cpm) and fold-change data are given in Supplementary Data sets S4 and S5. A GO analysis was performed using DAVID (Huang et al. 2009; Sherman et al. 2022). Since the GO analysis using tomato annotation gave poor results, the tomato genes were first annotated to their Arabidopsis homologs and a GO analysis was then performed using the Arabidopsis annotations.

### Accession numbers

Sequence data for genes used in this study can be found in the Sol Genomics Network under the following accession numbers: *SlPIF1a* (*Solyc09g063010*), *SlPIF1b* (*Solyc06g008030*), *SlPIF3* (*Solyc01g102300*), *SlPIF4* (*Solyc07g043580*), *SlPIF7a* (*Solyc03g115540*), *SlPIF7b* (*Solyc06g069600*), *SlPIF8a* (*Solyc01g090790*), and *SlPIF8b* (*Solyc10g018510*). RNA-seq (GSE267088) has been deposited into the Gene Expression Omnibus: https://www.ncbi.nlm.nih.gov/geo/query/acc.cgi?acc=GSE267088

## Supplementary Material

koaf048_Supplementary_Data

## Data Availability

The data underlying this article are available in the article and in its online supplementary material.

## Supplementary data

The following materials are available in the online version of this article.

**Supplementary Figure S1.** CRISPR-edited *SlPIF4*, *SlPIF7a*, *SlPIF7b*, and *SlPIF8a* genes in tomato.

**Supplementary Figure S2.** Expression of *SlPIF*s in response to low R/FR.

**Supplementary Figure S3.** Roles of SlPIFs, phyB1, and phyB2 in response to low R/FR ratios and elevated ambient temperatures in tomato.

**Supplementary Figure S4.** SlPIF8a is the major PIF that regulates the response to low R/FR in tomato.

**Supplementary Figure S5.** The *slpif4* mutants in an ID tomato background.

**Supplementary Figure S6.**  *SlPIF8b* does not play a role in the response to low R/FR.

**Supplementary Figure S7.** SlPIFs regulate the response to low R/FR in the cotyledons, first leaf, hypocotyl, and epicotyl.

**Supplementary Figure S8.** Low R/FR regulates cell wall-modifying genes in an *SlPIF*-dependent manner.

**Supplementary Figure S9.** Low R/FR regulates hormone biosynthesis genes in an SlPIFs-dependent manner.

**Supplementary Figure S10.** GO analysis of genes regulated by both low R/FR and SlPIFs in the cotyledons, first leaf, hypocotyl, and epicotyl.

**Supplementary Figure S11.** Spectral distributions of the light used in the white-light and low-R/FR conditions.

**Supplementary Table S1.** Primers used in this work.

**Supplementary Data Set 1.** List of genes present in the heatmaps in Fig. 4 and Supplementary Fig. S7.

**Supplementary Data Set 2.** List of genes present in the heatmaps in Fig. 5.

**Supplementary Data Set 3.** List of genes and GO terms present in Supplementary Fig. 10.

**Supplementary Data Set 4.** Complete RNA-seq data for the WT time course, including fold change, normalized cpm, and HTSeqcounts.

**Supplementary Data Set 5.** Complete RNA-seq data for WT, *slpif8a,* and *slpifq*, including fold change, normalized cpm, and HTSeqcounts.

**Supplementary Data Set 6.** Summary of statistical tests.

**Supplementary File 1.** Phylogenetic tree file.

**Supplementary File 2.** Alignments of the PIF proteins used for constructing the phylogenetic tree.

## Dive Curated Terms

The following phenotypic, genotypic, and functional terms are of significance to the work described in this paper:

HFR1 Gramene: AT1G02340
HFR1 Araport: AT1G02340

PIL1 Gramene: AT2G46970
PIL1 Araport: AT2G46970

ACS3 Gramene: AT5G28360
ACS3 Araport: AT5G28360

BAS1 Gramene: AT2G26710
BAS1 Araport: AT2G26710

CKX5 Gramene: AT1G75450
CKX5 Araport: AT1G75450

EXPA5 Gramene: AT3G29030
EXPA5 Araport: AT3G29030

NCED3 Gramene: AT3G14440
NCED3 Araport: AT3G14440

XTH33 Gramene: AT1G10550
XTH33 Araport: AT1G10550

PIF3 Gramene: AT1G09530
PIF3 Araport: AT1G09530

GA20ox2 Gramene: AT5G51810
GA20ox2 Araport: AT5G51810

PHYB Gramene: AT2G18790
PHYB Araport: AT2G18790

PIF7 Gramene: AT5G61270
PIF7 Araport: AT5G61270

PIF5 Gramene: AT3G59060
PIF5 Araport: AT3G59060

SlPIF7a Gramene: Solyc03g115540
SlPIF7a Araport: Solyc03g115540

SlPIF1a Gramene: Solyc09g063010
SlPIF1a Araport: Solyc09g063010

SlPIF1b Gramene: Solyc06g008030
SlPIF1b Araport: Solyc06g008030

SlPIF3 Gramene: Solyc01g102300
SlPIF3 Araport: Solyc01g102300

SlPIF4 Gramene: Solyc07g043580
SlPIF4 Araport: Solyc07g043580

SlPIF7b Gramene: Solyc06g069600
SlPIF7b Araport: Solyc06g069600

SlPIF8a Gramene: Solyc01g090790
SlPIF8a Araport: Solyc01g090790

SlPIF8b Gramene: Solyc10g018510
SlPIF8b Araport: Solyc10g018510

GSE Gramene: GSE267088
GSE Araport: GSE267088
