# Supplementary material for: Species-specific PHYTOCHROME-INTERACTING FACTOR utilization in the plant morphogenetic response to environmental stimuli
Source: Plant Cell. 2025 Mar 14;37(5):koaf048. doi: 10.1093/plcell/koaf048 (PMC12070396; doi:10.1093/plcell/koaf048)
Supplement: koaf048_Supplementary_Data [file koaf048_supplementary_data.zip › Supplementary Figures.pdf]

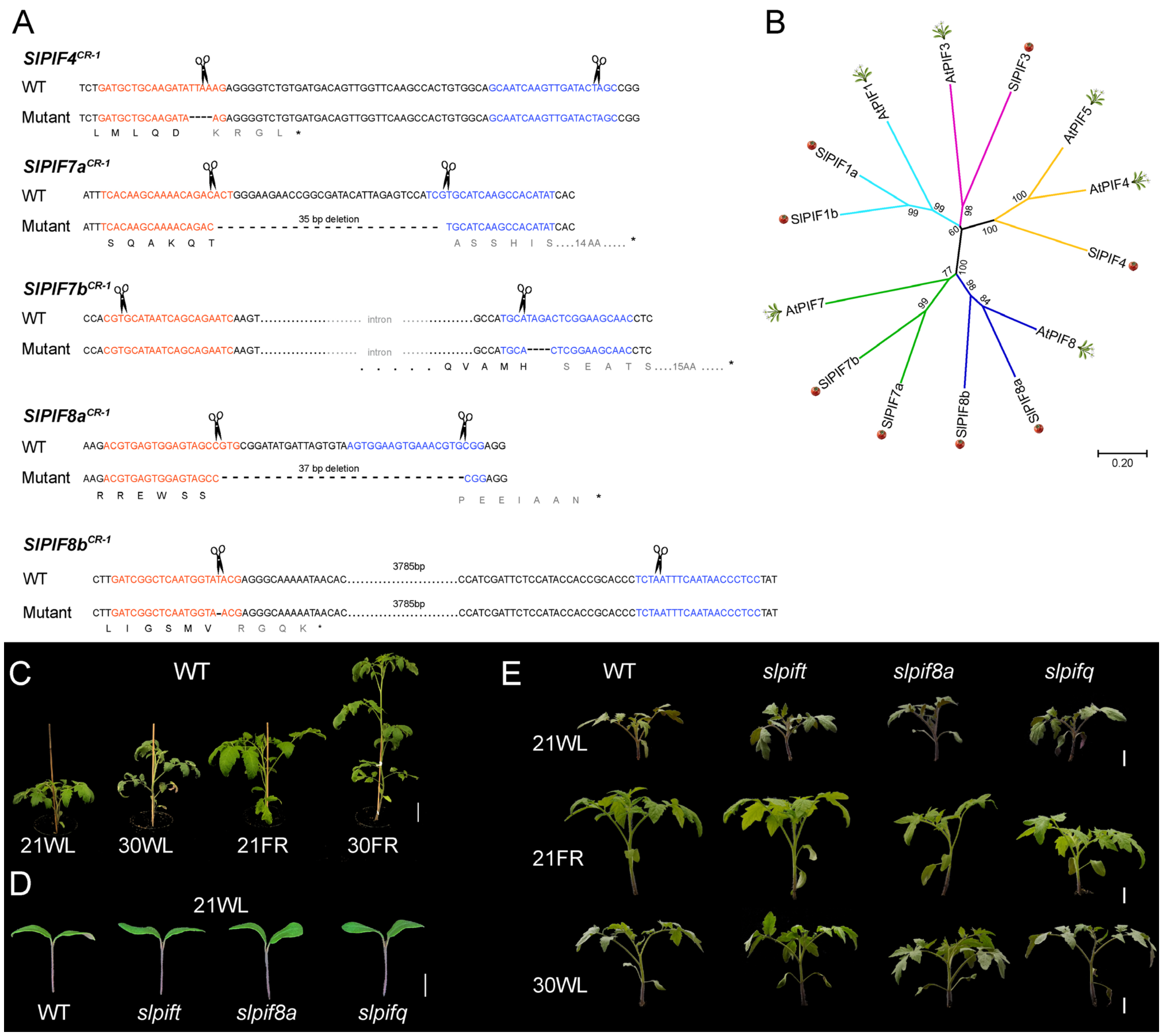

Supplementary Figure S1: CRISPR-edited *SlPIF4*, *SlPIF7a*, *SlPIF7b*, and *SlPIF8a* genes in tomato (Supports Figures 1 and 2).

A. Sequences of wild-type (WT, top) and edited *slpif4*, *slpif7a*, *slpif7b*, *slpif8a,* and *slpif8b* (middle) along with the predicted change in the mutant protein sequence (bottom). In each gene sequence, the nucleotides in orange and blue represent the gRNA1 and gRNA2 binding sites, respectively. The dashed lines between nucleotides represent deletions (with the number of base pairs deleted). A dotted line between nucleotides represents a gap in the gene sequence. Scissors mark the predicted cutting sites in the WT sequence. Black amino acids represent the WT sequence and gray amino acids represent the expected change in the protein sequence resulting from the CRISPR-mediated change in the gene sequence. An asterisk indicates the newly formed stop codon.
B. Phylogenetic analysis of *Arabidopsis thaliana* and *Solanum lycopersicum* PIF proteins, using full-length sequences (see Supplementary Files S1 and S2). Numbers at nodes represent bootstrap frequencies. Scale bar = Distance scale.
C. Representative images of 37-day-old wild-type tomato, grown under LD conditions at 21WL for 9 days and then moved to LD conditions at **30WL** (~200 µmol m^{-2} s^{-1}), **21FR** (~200 µmol m^{-2} s^{-1}, R/FR = 0.6), or **30FR** (~200 µmol m^{-2} s^{-1}, R/FR = 0.6), or kept at **21WL** (~200 µmol m^{-2} s^{-1}) for 28 more days. Scale bar = 5 cm (applicable to all images).

D. Representative images of 9-day-old tomato seedlings grown under long-day conditions (LD, 16/8) at 21°C under white light (~200 μmol m^{-2} s^{-1}, **21WL**) for 9 days. Scale bar = 1 cm (applicable to all images).

E. Representative images of 21-day-old tomato seedlings of the indicated genotypes grown under LD conditions and 21WL for 9 days, followed by 12 days under the indicated conditions. Scale bar = 2 cm (applicable to all the images from the same growth condition).

In **C-E**, images were digitally extracted for comparison.

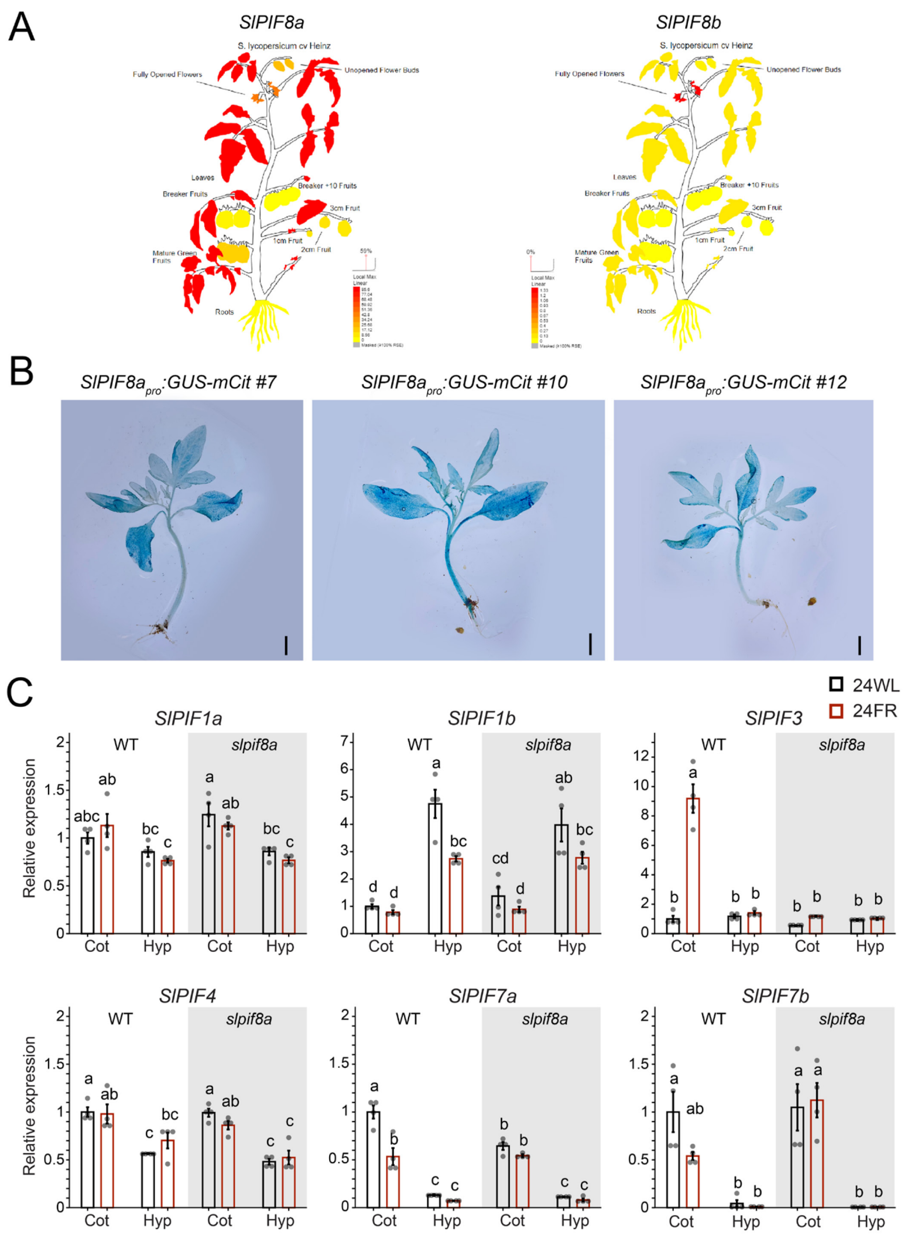

Supplementary Figure S2: Expression of *SlPIF*s in response to low R/FR (Supports Figure 1).

A. Screenshot from the tomato eFP browser (Waese et al. 2017; bar.utoronto.ca/efp2/Tomato/Tomato_eFPBrowser2) showing the expression levels of *SlPIF8a* and *SlPIF8b* in various parts of tomato plants.
B. Images of GUS-staining of tomato seedlings of three different lines carrying the *SlPIF8a*$_{pro}$*:GUS-mCitrine* reporter gene. Plants were grown under long-day conditions (LD, 16 h light/ 8 h dark) at 24°C under white light (**24WL**, ~200 µmol m^{-2} s^{-1}) for 12 days, followed by 24 h under LD + 24°C + white light supplemented with far-red light (**24FR**, ~200 µmol m^{-2} s^{-1}, R/FR = 0.6). Scale bar = 1 cm. Images were digitally extracted for comparison.
C. Relative expression of *SlPIF*s in the cotyledons and hypocotyls of 9-day-old wild-type (WT) and *slpif8a* seedlings grown under 24WL for 9 days and then either kept under the same conditions or moved to 24FR for 3 h. Gene expression was assayed using RT-qPCR relative to the reference gene *EXPRESS* and normalized to the expression in the wild-type cotyledons at 24WL. The average values of 4 biological replicates per condition ± SE are shown. Different letters denote statistical differences ($p < 0.05$) among samples, as assessed by one-way ANOVA and Tukey's HSD.

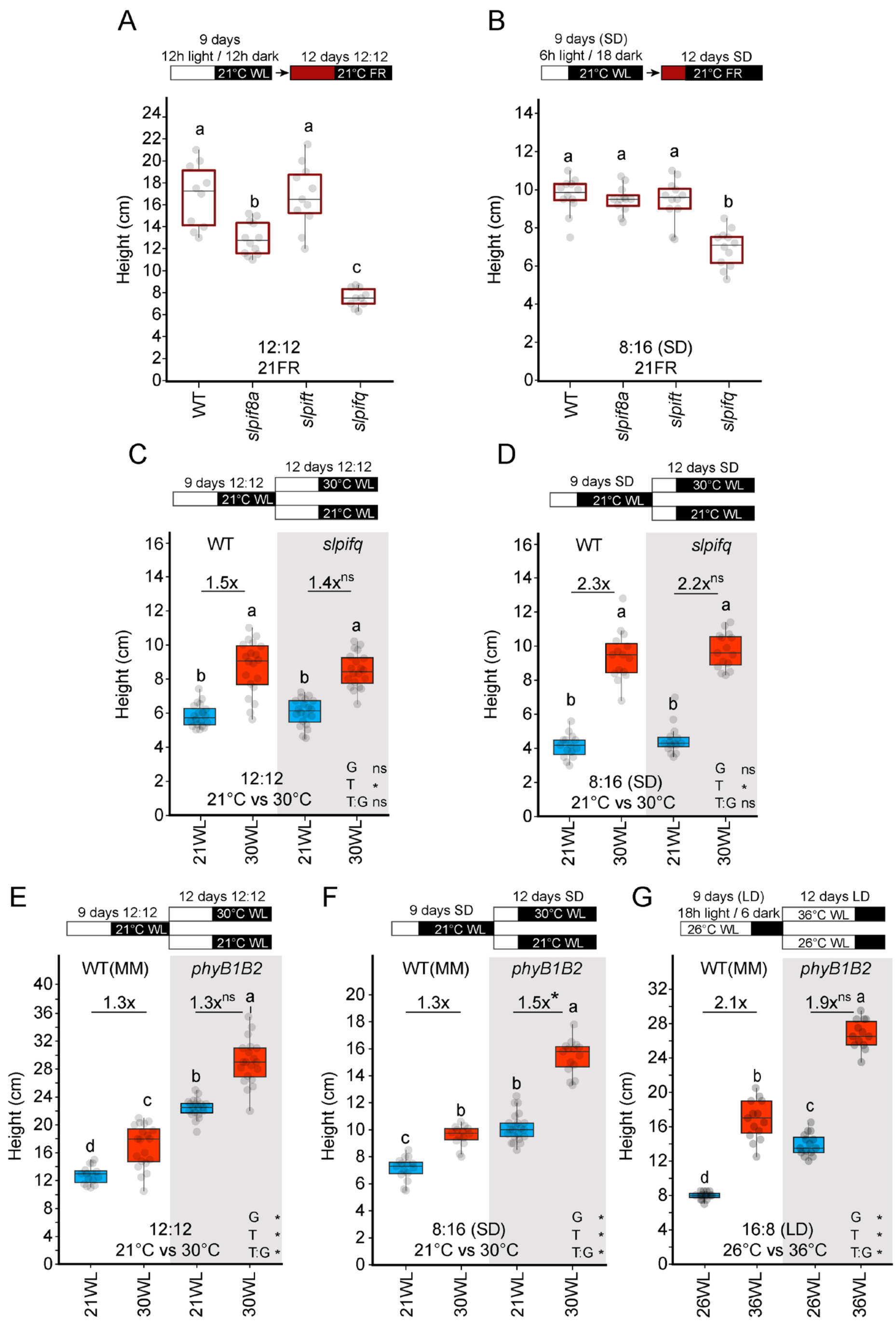

Supplementary Figure S3: Roles of SlPIFs, phyB1, and phyB2 in response to low R/FR ratios and elevated ambient temperatures in tomato (Supports Figure 1).

A, B. Plant height of 21-day-old wild-type and *slpif*-mutant seedlings grown under day natural conditions (12 h light / 12 h dark; 12:12, ~160 µmol m^{-2} s^{-1}, **A**) or short-day conditions (8 h light / 16 h dark; SD, ~200 µmol m^{-2} s^{-1}, **B**) at 21°C white light for 9 days, and then moved to (**A**) 12:12 or (**B**) SD conditions at 21°C supplemented with far-red light (21FR, R/FR = 0.6). n > 10 seedlings per sample.

C, D. Plant height of 21-day-old wild-type and *slpifq* mutant seedlings grown in (**C**) 12:12 (~160 µmol m^{-2} s^{-1}) or (**D**) 8:16 (SD, ~200 µmol m^{-2} s^{-1}) at 21°C in white light for 9 days, and then moved to the same light conditions at either 30°C (30WL) or 21°C (21WL). n > 15 seedlings per sample.

E, F. Plant height of 21-day-old wild-type and *phyB1B2* mutant seedlings grown in (**E**) 12:12 (~160 μmol m^{-2} s^{-1}) or (**F**) 8:16 (SD, ~200 μmol m^{-2} s^{-1}) at 21°C in white light for 9 days, and then moved to the same light conditions at either 30°C (30WL) or 21°C (21WL). $n > 14$ seedlings per sample.

G. Plant height of 21-day-old wild-type and *phyB1B2* mutant seedlings grown under long-day conditions (16 h light / 8 h dark; LD) at 26°C under white light (~200 μmol m^{-2} s^{-1}) for 9 days and then transferred to LD at either 36°C (36WL) or 26°C (26WL) under white light. $n > 13$ seedlings per sample.

Boxes indicate the first and third quartiles, whiskers indicate the minimum and maximum values, black lines within the boxes indicate the median values, and gray dots indicate the individual data points. Different letters denote statistical differences ($p < 0.05$) among samples, as assessed by one-way ANOVA (**A, B**) or two-way ANOVA (**C–G**) and Tukey's HSD. In **C–G**, the average fold change between the high and low temperatures is shown. Asterisks indicate significant differences ($p < 0.05$) based on Student's *t*-test or ANOVA. T, temperature treatment; G, genotype; T:G, the interaction between temperature treatment and genotype; ns, not significant.

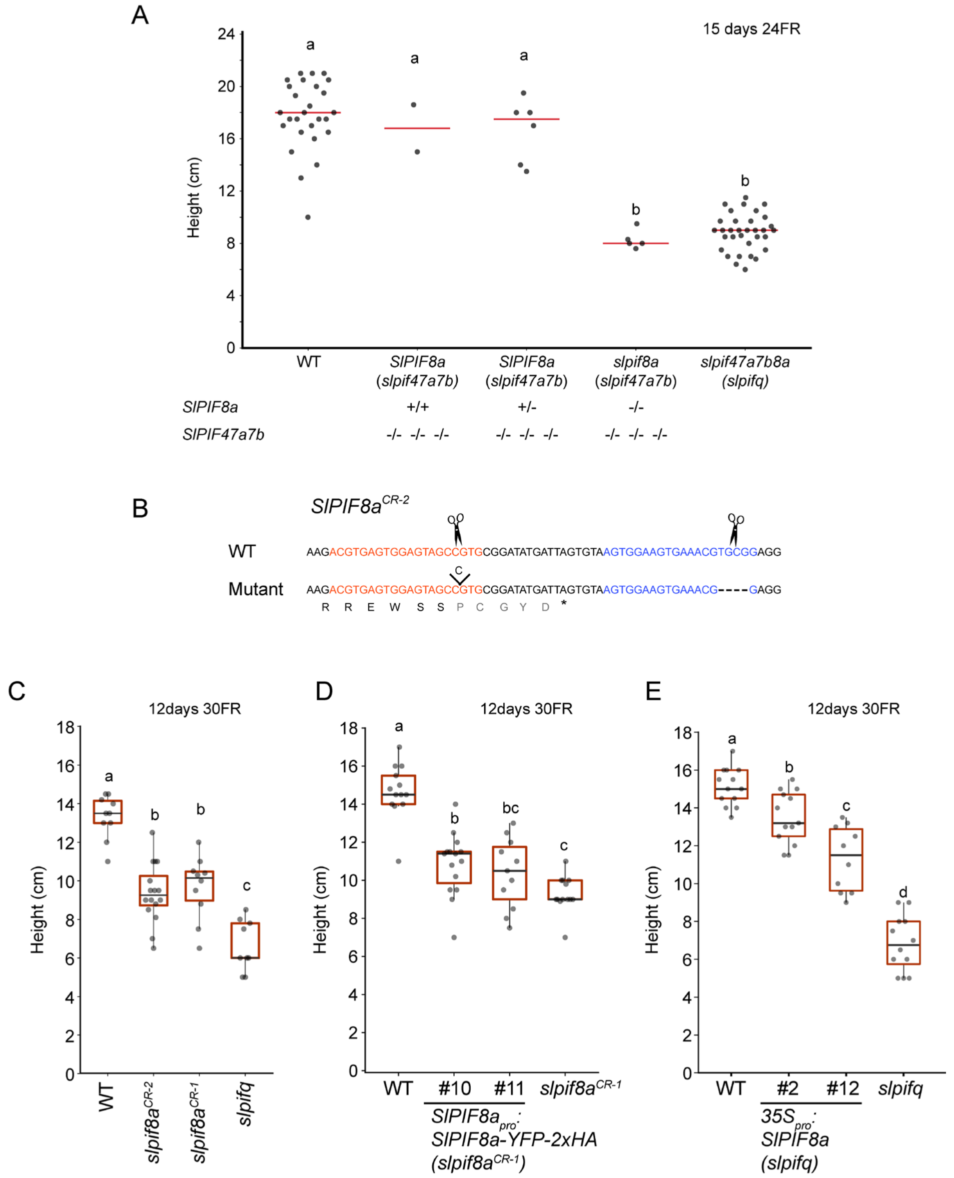

Supplementary Figure S4: SlPIF8a is the major PIF that regulates the response to low R/FR in tomato (Supports Figures 1 and 3).

A. Total height of 24-day-old WT, *slpifq*, and segregate plants; homozygous in *slpif4*, *slpif7a*, and *slpif7b*; and heterozygous in *slpif8a* as indicated (+/+ wild-type; +/- heterozygote; -/- mutant). Seedlings were grown at 24WL for 9 days and then moved to 24FR for 15 days. Different letters denote statistical differences ($p < 0.05$) among samples as assessed by one-way ANOVA and Tukey's HSD. Red lines indicate the median values and black dots mark individual seedlings. $n > 5$, except for *slpif47a7b* (*SlPIF8a* +/+) in which $n = 2$.
B. Sequences of WT (top) and *slpif8a*$^{CR\text{-}2}$ allele (bottom) with the inserted nucleotide highlighted. Nucleotides in orange and blue represent the gRNA1 and gRNA2 binding sites, respectively, and scissors indicate the predicted cutting site. Black amino acids indicate the wild-type sequence and gray amino acids indicate a predicted change in the protein sequence resulting from the CRISPR-mediated gene modification. A dashed line between nucleotides represents a deletion, and an asterisk indicates a newly formed stop codon. *slpif8a*$^{CR\text{-}1}$ allele (Supplementary Fig. S1A) was used for all experiments described in this paper, unless otherwise noted.

C-E. Total height of 21-day-old WT plants, *slpifq* plants, and two alleles of *slpif8a* plants (**C**). (**D**) Rescue lines of *slpif8a* expressing *SlPIF8a*$_{pro}$*:SlPIF8a-YFP-2xHA*, and (**E**) *slpifq* rescue lines expressing *35S*$_{pro}$*:SlPIF8a*. The seedlings were grown at 21WL for 9 days and then moved to 30FR for 12 more days. $n > 9$ seedlings per sample. Different letters denote statistical differences ($p < 0.05$) among samples, as assessed by one-way ANOVA and Tukey's HSD. Boxes indicate the first and third quartiles, whiskers indicate the minimum and maximum values, black lines within the boxes indicate the median values, and gray dots indicate the individual data points.

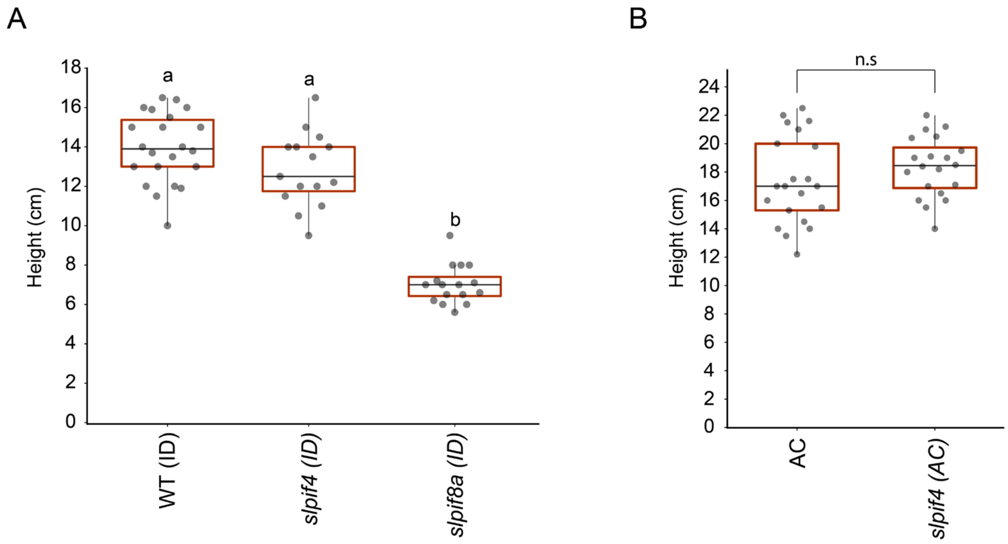

Supplementary Figure S5: The *slpif4* mutants in an indeterminate tomato background (Supports Figure 1).

A. Total height of 21-day-old wild-type (M82), *slpif4*, and *slpif8a* indeterminate (ID) tomato plants. $n > 15$ seedlings per sample. Different letters denote statistical differences ($p < 0.05$) among samples, as assessed by one-way ANOVA and Tukey's HSD.
B. Total height of the 21-day-old tomato *slpif4* mutant and its indeterminate background, corresponding to the Ailsa Craig (AC) background. $n > 20$ seedlings per sample. ns, not significant based on Student's *t*-test ($p < 0.05$).

In **A** and **B**, the seedlings were grown at 21WL for 9 days and then moved to 30FR for 12 more days. Boxes indicate the first and third quartiles, whiskers indicate the minimum and maximum values, black lines within the boxes indicate the median values, and gray dots indicate the individual data points.

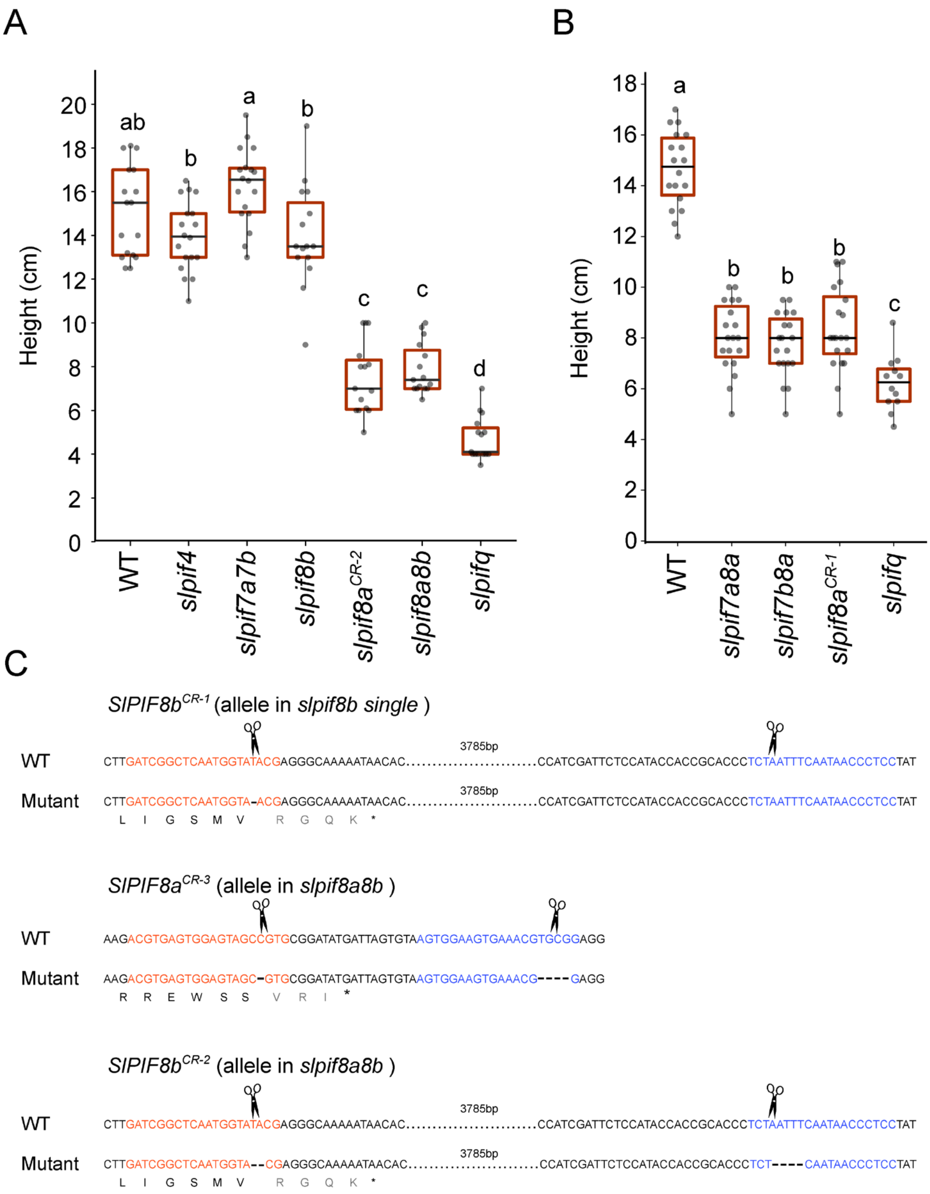

Supplementary Figure S6: *SlPIF8b* does not play a role in the response to low R/FR (Supports Figure 1).

A, B. Total height of 21-day-old wild-type plants and the indicated *slpif* mutants. The seedlings were grown at 21WL for 9 days and then moved to 30FR for 12 more days. *n* > 12 seedlings per sample. Different letters denote statistical differences ($p < 0.05$) among samples, as assessed by one-way ANOVA and Tukey's HSD. Boxes indicate the first and third quartiles, whiskers indicate the minimum and maximum values, black lines within the boxes indicate the median values, and gray dots indicate the individual data points.

C. Sequences of WT (top) and edited alleles *slpif8b*$^{CR-1}$ (also present in Supplementary Fig. S1A) *slpif8a*$^{CR-3}$, and *slpif8b*$^{CR-2}$ (middle) along with the predicted change in the mutant protein sequence (bottom). In each gene sequence, the nucleotides in orange and blue represent the gRNA1 and gRNA2 binding sites, respectively, and scissors indicate the predicted cutting site. A dotted line between nucleotides represents a gap in the gene sequence. Black amino acids indicate the WT sequence and gray amino acids indicate the predicted change in the protein sequence resulting from the CRISPR-mediated gene modification. A dashed line between nucleotides represents a deletion, and an asterisk indicates a newly formed stop codon.

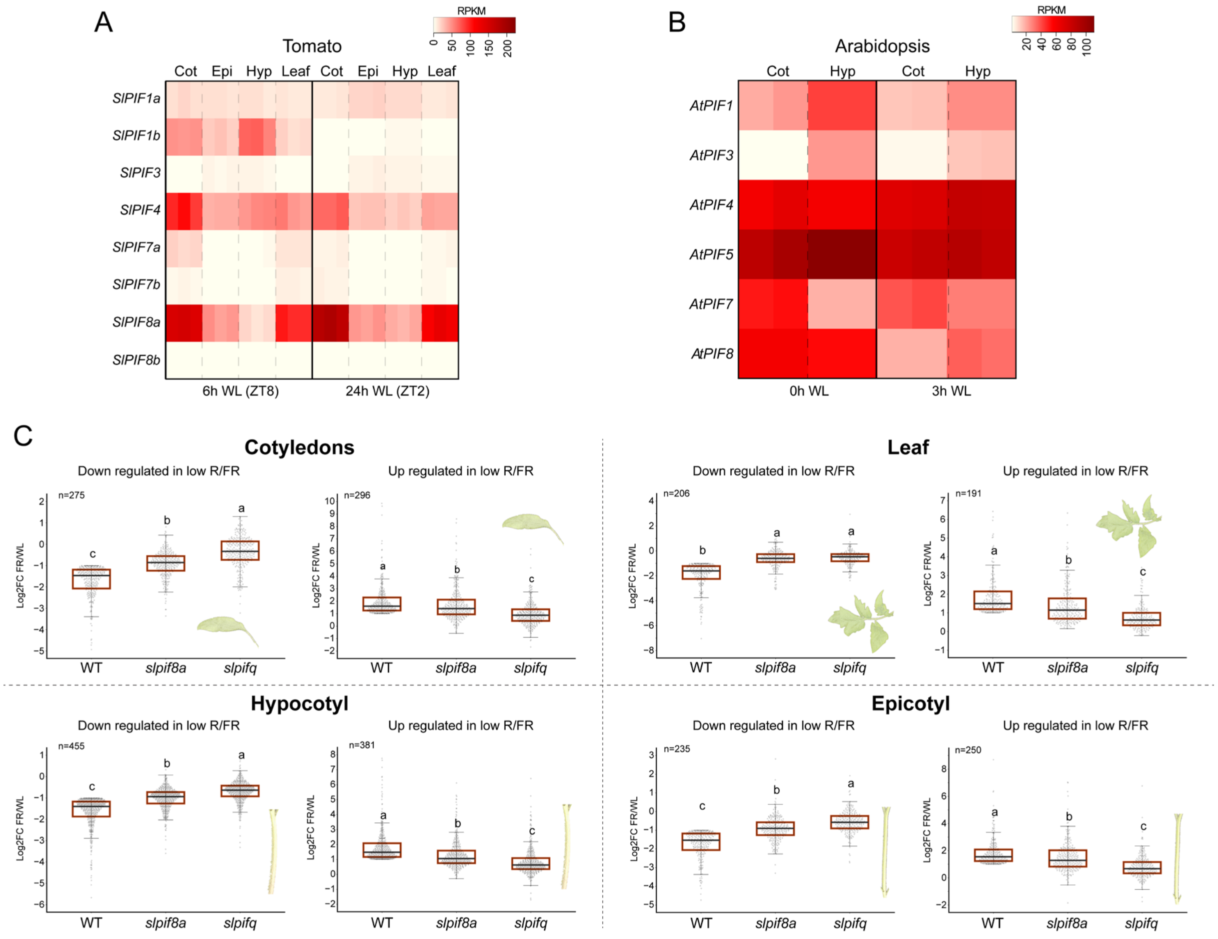

Supplementary Figure S7: SlPIFs regulate the response to low R/FR in the cotyledons, first leaf, hypocotyl, and epicotyl (Supports Figure 4).

A. Tissue-specific expression levels of tomato *SlPIF* genes in wild-type seedlings exposed to white light for the indicated period of time. The color scale represents the reads per kilobase per million mapped reads (RPKM).
B. Tissue-specific expression levels of Arabidopsis *At-PIF* genes in wild-type seedlings at the beginning of the experiment (0 h) and after 3 h of exposure to white light. The color scale represents RPKM. Raw data taken from Kohnen et al. 2016.
C. Tissue-specific relative expression of genes downregulated (left) or upregulated (right) in wild-type seedlings exposed to 24FR for 6 h, corresponding to the heatmap presented in **Fig. 4D**. Log2FC: Log2 Fold Change relative to 24WL for each tissue specifically and for each genotype. Different letters denote statistical differences ($p < 0.05$) within time points, as assessed by one-way ANOVA and Tukey's HSD. Boxes indicate the first and third quartiles and whiskers indicate the minimum and maximum values. The black lines within the boxes indicate the median values, and gray dots indicate individual genes. Sample sizes (*n*) are indicated in the top left corner of each plot.

For **A** and **B**, see Supplementary Dataset S1; Cot, cotyledons; Leaf, first leaf; Hyp, hypocotyl; Epi, epicotyl.

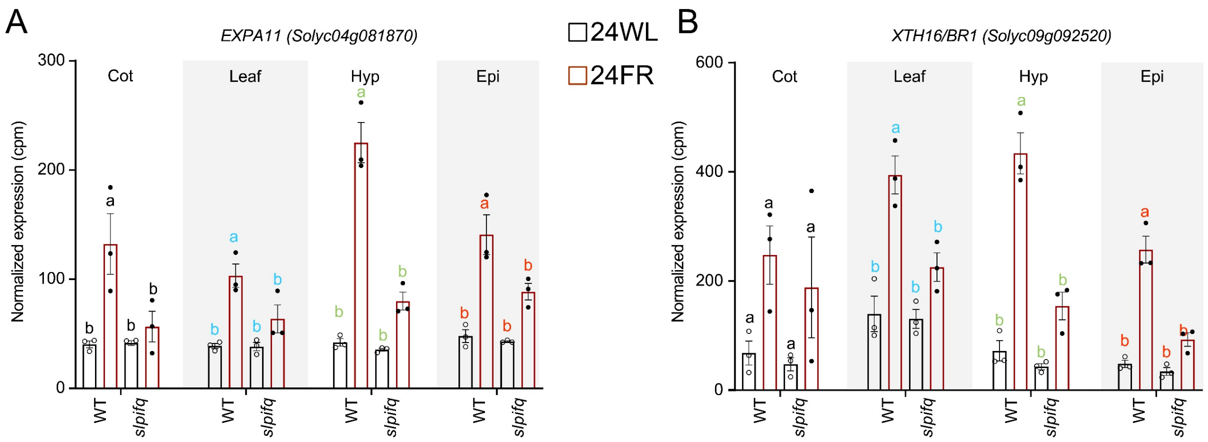

Supplementary Figure S8: Low R/FR regulates cell wall-modifying genes in an *SlPIF*-dependent manner (Supports Figure 4).

Expression levels (derived from the RNA-seq data) of tomato *EXPA11* (*Solyc04g081870*, **A**) and *XTH16/BR1* (*Solyc09g092520*, **B**). The expression levels in the indicated organs (Cot, cotyledons; Leaf, first leaf; Hyp, hypocotyl; Epi, epicotyl) of wild-type and *slpifq* plants grown as described in **Fig. 1H**, followed by 6 h of 24FR (red bars) or 24WL (black bars) are shown. Data are presented as normalized counts per million mapped reads (cpm). The average values of 3 biological replicates per condition ± SE are presented. Different letters denote statistical differences ($p < 0.05$) among samples, as assessed by one-way ANOVA and Tukey's HSD for each organ separately.

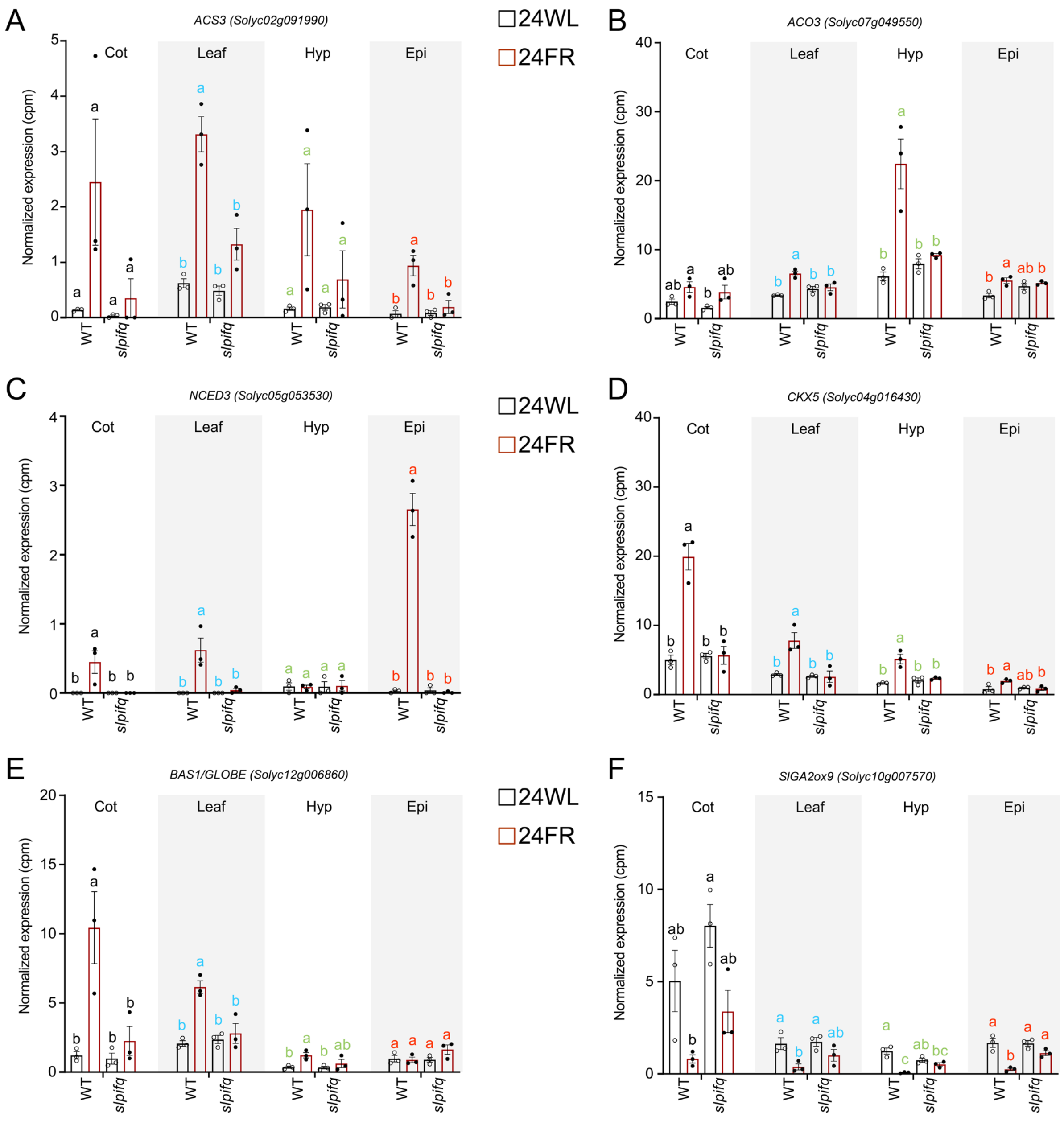

Supplementary Figure S9: Low R/FR regulates hormone biosynthesis genes in an SlPIFs-dependent manner (Supports Figure 4).

Expression levels (derived from the RNA-seq data) of tomato *ACS3* (*Solyc02g091990*, **A**), *ACO3* (*Solyc07g049550*, **B**), *NCED3* (*Solyc05g053530*, **C**), *CKX5* (*Solyc04g016430*, **D**), *BAS1/GLOBE* (*Solyc12g006860*, **E**), and *SlGA2ox9* (*Solyc10g007570*, **F**). The expression levels in the indicated organs (Cot, cotyledons; Leaf, first leaf; Hyp, hypocotyl; Epi, epicotyl) of wild-type and *slpifq* plants, grown as described in Fig. 1H followed by 6 h of 24FR (red bars) or 24WL (black bars) are shown. Data are presented as normalized counts per million mapped reads (cpm). The average values of 3 biological replicates per condition ± SE are presented. Different letters denote statistical differences ($p < 0.05$) among samples, as assessed by one-way ANOVA and Tukey's HSD for each organ separately.

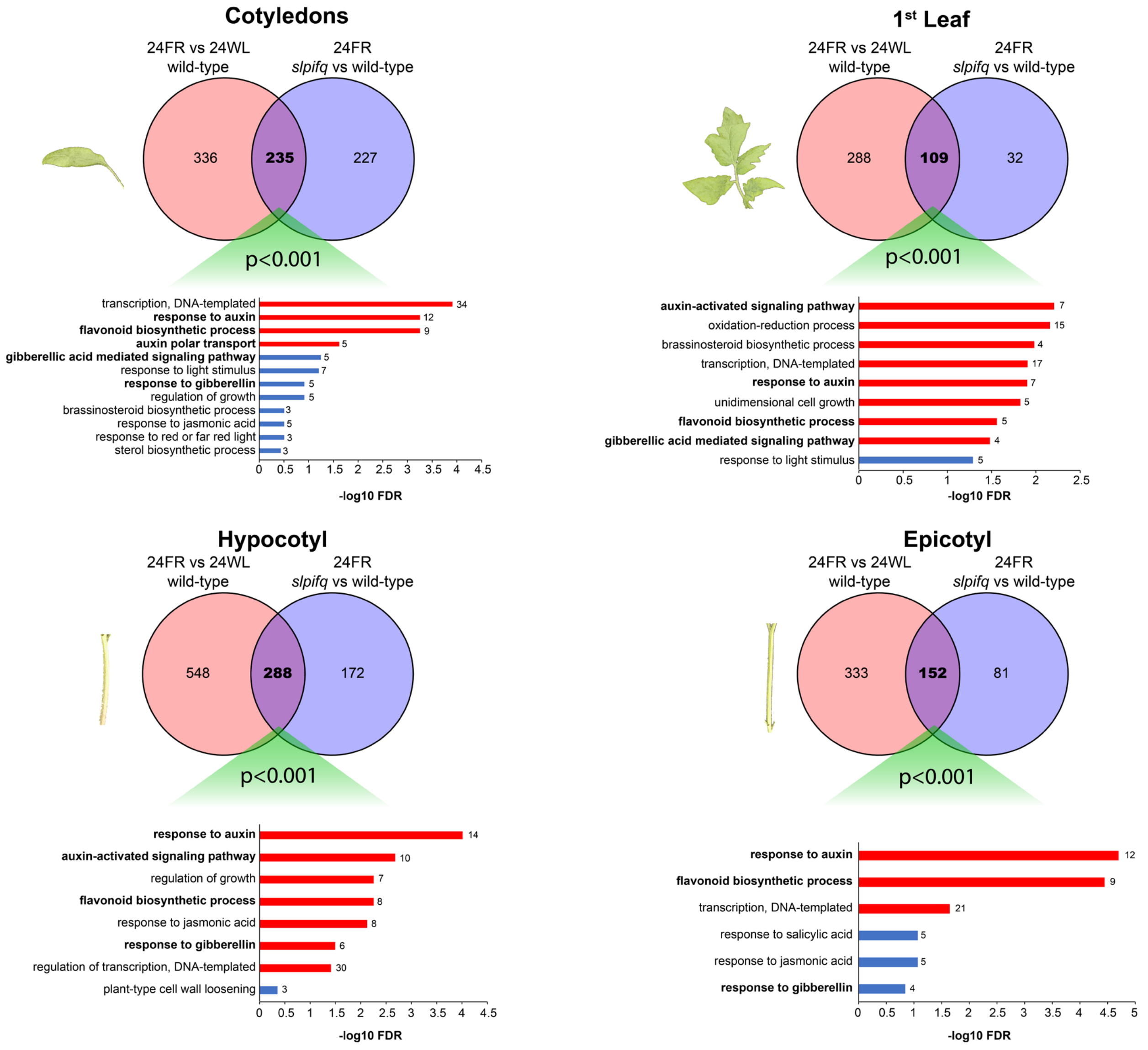

Supplementary Figure S10: Gene ontology (GO) analysis of genes regulated by both low R/FR and SlPIFs in the cotyledons, first leaf, hypocotyl, and epicotyl (Supports Figures 4 and 5).

Venn diagrams comparing the tissue-specific differentially expressed genes (FDR < 0.05 and FC > 1 or < -1) in wild-type at 24FR versus 24WL (red) or wild-type versus *slpifq* (blue) or both. The number of genes in each group is shown. Fisher's exact test was used to test for overrepresented gene overlap. For a list of genes in each group, see Supplementary Dataset S3. The GO enrichment of genes expressed differently in 24FR versus 24WL and *slpifq* versus wild-type is presented at the bottom for each tissue. The lengths of the bars indicate the - log10 FDR, and the number next to the bar shows the number of genes with the presented GO term. Red bars: FDR < 0.05; blue bars: FDR > 0.05; and $p < 0.05$. For the full results of the GO analysis, see Supplementary Dataset S3.

A

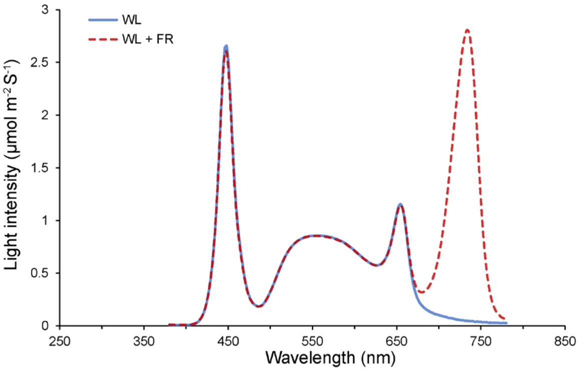

WL

B	R	FR	R:FR	PAR
70	57	4	14.13	202

WL+FR

B	R	FR	R:FR	PAR
69	64	106	0.60	208

B

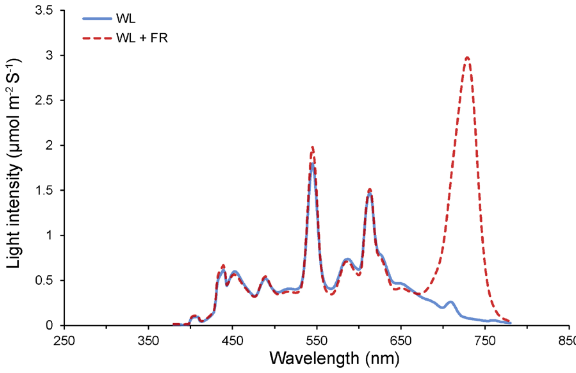

WL

B	R	FR	R:FR	PAR
36	57	8	7.20	159

WL+FR

B	R	FR	R:FR	PAR
36	62	105	0.60	163

C

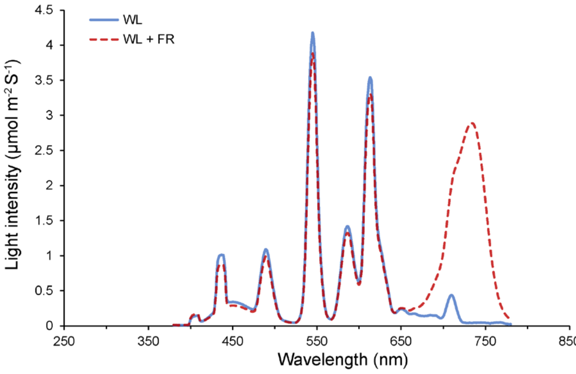

WL

B	R	FR	R:FR	PAR
43	74	8	8.75	205

WL+FR

B	R	FR	R:FR	PAR
38	83	135	0.62	204

Supplementary Figure S11: Spectral distributions of the light used in the white-light and low-R/FR conditions (Supports Figures 1-5).

Spectral distributions, measured with a LI-COR LI-180 spectrometer, of white light (WL, blue line) and white light supplemented with far-red light (WL + FR, red dashed line). The spectrum shown in **A** was used for all experiments, except for those described in Supplementary Fig. S3 A-F, for which the spectrums shown in **B** and **C** were used. The spectrum shown in **B** was used for a 12 h light / 12 h dark (12:12) setting, while the spectrum shown in **C** was used for an 8 h light / 16 h dark (short-day, SD) setting.

Supplementary Table S1: Primers used in this work.

Primer name	Sequence 5' >>> 3'	Purpose	Use
AttB4_SlPIF8a_p_F	GGGGACAACTTTGTATAGAAAAGTTGYYAAGAGATGGAGCGACACCAA	Cloning *SlPIF8a* promoter to pDONR P4-P1	Transform into tomato
AttB1_SlPIF8a_p_R	GGGGACTGCTTTTTTGTACAAACTTGYATCGAAGGTAAATAAGAGGGAGGT		
AttB1_SlPIF8a_F	GGGGACAAGTTTGTACAAAAAAGCAGGCTGCATGAATCAGTGTGTACCGAGTTG	Cloning SlPIF8a to pDONR P1-P2	
AttB2_SlPIF8a_R	GGGGACCACTTTGTACAAGAAAGCTGGGTCATTTTTAGGGCCAGAGCCA		
SlPIF4_gRNA_1	TGTGGTCTCAATTGCAATCAAGTTGATACTAGCGTTTTAGAGCTAGAAATAGCAAG	Cloning SlPIF4 gRNAs with universal primer	
SlPIF4_gRNA_2	TGTGGTCTCAATTGATGCTGCAAGATATTAAAGGTTTTAGAGCTAGAAATAGCAAG		
SlPIF7a_gRNA_1	TGTGGTCTCAATTGATATGTGGCTTGATGCACGAGTTTTAGAGCTAGAAATAGCAAG	Cloning SlPIF7a gRNAs with universal primer	
SlPIF7a_gRNA_2	TGTGGTCTCAATTGTCACAAGCAAAACAGACACTGTTTTAGAGCTAGAAATAGCAAG		
SlPIF7b_gRNA_1	TGTGGTCTCAATTGATTCTGCTGATTATGCACGGTTTTAGAGCTAGAAATAGCAAG	Cloning SlPIF7b gRNAs with universal primer	
SlPIF7b_gRNA_2	TGTGGTCTCAATTGTTGCTTCCGAGTCTATGCAGTTTTAGAGCTAGAAATAGCAAG		
SlPIF8a_gRNA_1	TGTGGTCTCAATTGAGTGGAAGTGAAACGTGCGGGTTTTAGAGCTAGAAATAGCAAG	Cloning SlPIF8a gRNAs with universal primer	
SlPIF8a_gRNA_2	TGTGGTCTCAATTGACGTGAGTGGAGTAGCCGTGGTTTTAGAGCTAGAAATAGCAAG		
SlPIF8b_gRNA_1	TGTGGTCTCAATTGATCGGCTCAATGGTATACGGTTTTAGAGCTAGAAATAGCAAG	Cloning SlPIF8b gRNAs with universal primer	
SlPIF8b_gRNA_2	TGTGGTCTCAATTGGAGGGTTATTGAAATTAGAGTTTTAGAGCTAGAAATAGCAAG		
gRNA_universal_primer	TGTGGTCTCAAGCGTAATGCCAACTTTGTAC	PCR with gRNA primers	
SlPIF4_RT_F	CAAGGCGCTTCTCAGCCTAT	SlPIF4	RT-qPCR
SlPIF4_RT_R	GGATGCGGTAACTGCTGAGT		
SlPIF8a_RT_F	CCCCGGACTTCCTTCCTTTC	SlPIF8a	
SLPIF8a_RT_R	AAGGCGCGGAGTTTGAGTTA		
SlPIF7a_RT_F	TGGTCTTGGCATGGGTATGG	SlPIF7a	
SlPIF7a_RT_R	CACTGGCAGGGCTAACAGAA		
SlPIF7b_RT_F	GCGCTCCCCTCATTTATCCA	SlPIF7b	
SlPIF7b_RT_R	TTTGGGGCTGATGGATTCGG		
SlPIF3_RT_F	AGCTGCCCACAGAATCAGAC	SlPIF3	
SlPIF3_RT_R	TGGGACCAGCTTCATTTCCG		
SlPIF1a_RT_F	CGAAGGTCAGAATGAGGATG	SlPIF1a	
SLPIF1a_RT_R	AGCACGAGATCTCTTTGTGGA		
SlPIF1b_RT_F	GCTGATGCAAGGAAGCAAGT	SlPIF1b	
SlPIF1b_RT_R	CTGTCTCGGCGTTTCCTTT		
Express_F	TGGGTGTGCCTTTCTGAATG	Express, control gene	
Express_R	GCTAAGAACGCTGGACCTAATG		

SlPIF7a det- F	CATGGGAAAAAGGGCAGTTA	Genotype SlPIF7a	Genotyping
SlPIF7a det- R	CAGCACCCCAGTTTTCAGAT		
SlPIF7b det- F	TCACCAAAAACAAGAACAACAA	Genotype SlPIF7b	
SlPIF7b det- R	TTGCCTGATTTTGTCCATCA		
SlPIF4 det- F	CAATGCCACCTCCAAGATTT	Genotype SlPIF4	
SlPIF4 det- R	TTCAAATGTGTCTCGGTCCA		
SlPIF8a det- F	ACCTCAAATTCGGACAACCA	Genotype SlPIF8a	
SlPIF8a det- R	GTCGTACCAAATTCTCTATCAAACG		
SlPIF8b det- F1	GTGACGAGGGTGGTGATGAT	Genotype SlPIF8b	
SlPIF8b det- R1	TGTGCTGCATGGGAATCATA		
SlPIF8b det- F2	TTTTGTTTAAGCAGACTGATACGG		
SlPIF8b det- R2	TTGTACCTGTGATAATTGGCGTA		
CAS9 F	CGCTAATCTTGCAGGTAGCC	Genotype for CAS9-free plants	
CAS9 R	TGCCAGCTCGTTACCTTTCT		
SP F	TTACTCCGTCTGTCTGTCCTAAT	Genotype for SP gene + BstNI enzyme	
SP R	CGTCTAATCAACTGTACGACAACA		

References

1. **Kohnen, M.V., Schmid-Siegert, E., Trevisan, M., Petrolati, L.A., Senechal, F., Muller-Moule, P., Maloof, J., Xenarios, I., and Fankhauser, C.** (2016). Neighbor Detection Induces Organ-Specific Transcriptomes, Revealing Patterns Underlying Hypocotyl-Specific Growth. *Plant Cell* **28,** 2889-2904.

2. **Waese, J., Fan, J., Pasha, A., Yu, H., Fucile, G., Shi, R., Cumming, M., Kelley, L.A., Sternberg, M.J., Krishnakumar, V., Ferlanti, E., Miller, J., Town, C., Stuerzlinger, W., and Provart, N.J.** (2017). ePlant: Visualizing and Exploring Multiple Levels of Data for Hypothesis Generation in Plant Biology. *Plant Cell* **29,** 1806-1821.
